# HeLa cell response proteome alterations induced by mammalian reovirus T3D infection

**DOI:** 10.1186/1743-422X-10-202

**Published:** 2013-06-21

**Authors:** Kevin M Coombs

**Affiliations:** 1 Department of Medical Microbiology, Faculty of Medicine, University of Manitoba, Winnipeg, MB R3E 0J9, Canada; 2 Manitoba Center for Proteomics and Systems Biology, University of Manitoba, Room 799 John Buhler Research Centre, 715 McDermot Avenue, Winnipeg, MB R3E 3P4, Canada; 3 Manitoba Institute of Child Health, University of Manitoba, Winnipeg, MB R3E 3P4, Canada

**Keywords:** RNA virus, Virus infection, Host cell alterations, Proteomics, Mass spectrometry, Liquid chromatography, Bioinformatics

## Abstract

**Background:**

Cells are exposed to multiple stressors that induce significant alterations in signaling pathways and in the cellular state. As obligate parasites, all viruses require host cell material and machinery for replication. Virus infection is a major stressor leading to numerous induced modifications. Previous gene array studies have measured infected cellular transcriptomes. More recently, mass spectrometry-based quantitative and comparative assays have been used to complement such studies by examining virus-induced alterations in the cellular proteome.

**Methods:**

We used SILAC (stable isotope labeling with amino acids in cell culture), a non-biased quantitative proteomic labeling technique, combined with 2-D HPLC/mass spectrometry and reciprocal labeling to identify and measure relative quantitative differences in HeLa cell proteins in purified cytosolic and nuclear fractions after reovirus serotype 3 Dearing infection. Protein regulation was determined by z-score analysis of each protein’s label distribution.

**Results:**

A total of 2856 cellular proteins were identified in cytosolic fractions by 2 or more peptides at >99% confidence and 884 proteins were identified in nuclear fractions. Gene ontology analyses indicated up-regulated host proteins were associated with defense responses, immune responses, macromolecular binding, regulation of immune effector processes, and responses to virus, whereas down-regulated proteins were involved in cell death, macromolecular catabolic processes, and tissue development.

**Conclusions:**

These analyses identified numerous host proteins significantly affected by reovirus T3D infection. These proteins map to numerous inflammatory and innate immune pathways, and provide the starting point for more detailed kinetic studies and delineation of virus-modulated host signaling pathways.

## Background

The cellular proteome (the total protein repertoire, which includes how each protein may be co-translationally or post-translationally modified) is affected by numerous stresses, including infection by viruses. Numerous previous microarray studies have determined how cellular transcriptomes respond after virus infection (see for example: [1,2]). However, since mRNA levels cannot provide complete information about levels of protein synthesis or the types and degrees of post-translational modifications, there frequently is little concordance between microarray and protein data [3-5]. Therefore, quantitative and comparative proteomic analyses are also being used to provide additional information about host alterations to virus infection (reviewed in: [6,7]). Commonly used methods include 2-dimensional difference in gel electrophoresis (2D-DIGE (see for examples: [8,9]), isotope coded affinity tags (ICAT; [10,11]), isobaric tags for relative and absolute quantitation (iTRAQ; [12,13]), and stable isotope labeling by amino acids in cell culture (SILAC; [14-18]). The SILAC method involves labeling cells with “light” (normal; **L**) and “heavy” (**H**) isotopic forms of amino acids. Advantages of this particular technique include: experimental set-up is relatively simple, **L** and **H** samples are mixed together early in this process, thereby reducing sample-to-sample variability, and, if ^12^C_6_-Lys and ^12^C_6_^14^N_4_-Arg (**L**), and ^13^C_6_-Lys and ^13^C_6_^15^N_4_-Arg (**H**) amino acids are used, virtually every tryptic peptide should contain a labeled amino acid, thereby providing increased protein coverage. Indeed, several early comparative studies suggested SILAC provided more identifications than the other commonly used methods (reviewed in [7]). We have been using SILAC to measure comparative proteomic alterations induced by influenza virus in A549 [19] and in normal human bronchial epithelial (NHBE) airway cells [20]. We have also begun similar analyses with reovirus-infected cells, focusing initially upon reovirus serotype T1L-infected HEK-293 cells [21].

The mammalian reoviruses (MRV) are non-enveloped viruses with a genome that consists of 10 segments of double-stranded (ds)RNA. The dsRNA genome is enclosed in a double-layered concentric protein capsid composed of 8 viral structural proteins. For reviews, see [22-24]. MRV is the prototype member of the Orthoreovirus genus in the family *Reoviridae*. The Ortheoreoviruses include nonfusogenic MRV and fusogenic avian reovirus. The Reoviridae family also contains rotaviruses [25], orbiviruses [26], and at least 9 other genera, several of which can infect animals, insects and/or plants [23,24]. MRV infections are generally mild in humans but many of the other family members are highly pathogenic in their hosts. MRV currently consist of 4 identified serotypes, with each represented by a prototype strain: strain Lang (T1L) for serotype 1; strain Jones (T2J) for serotype 2, strain Dearing (T3D) for serotype 3 and strain Ndelle for serotype 4 [27]. The reoviruses have long served as models for understanding viral pathogenesis [22] and they have also been identified as potential oncolytic agents [28-30] because of their capacity to selectively kill cancer cells that contain activated Ras pathway and functional p53 [28,31].

MRV are capable of infecting a wide range of cells, including mouse L929 cells, often used for stock preparation and titration [32], and various human cells, including HEK-293 [21]. Numerous reovirus studies have also been performed in HeLa cells (see for examples: [33-36]). Global microarray analyses of MRV-infected cellular transcriptomes detected activation of numerous cellular genes, including many related to apoptosis [37-41]. These microarray assays have recently been complemented by quantitative and comparative proteomic analyses. For example, Li and colleagues recently demonstrated, using 2D-DIGE, that MRV-infected murine myocytes regulate several proteins, including heat shock proteins and interferon-response proteins [42]. We have also shown, using SILAC and 2D-HPLC/MS, that proteins involved in cell death, cell growth and proliferation, molecular transport, gene expression, and inflammatory response pathways are affected in MRV T1L-infected HEK-293 cells [21]. Similar pathways were also found regulated in a preliminary analysis of reovirus T3D-infected HeLa cells that concluded that inclusion of Proteominer® bead-based non-biased enrichment did not significantly improve proteomic coverage of unpurified cell extracts [43].

Thus, as part of an ongoing systematic delineation of reovirus-induced comparative host protein responses, we are examining how reoviruses T1L and T3D, two of the most commonly used MRV strains, affect various permissive cells. We have extended our previous T1L-infected HEK-293 cell study [21] to T3D-infected HEK-293 cells (Berard, in preparation). Our previous proteomic analysis of HeLa cells infected with T3D concluded that inclusion of Proteominer® bead-based non-biased enrichment did not significantly improve proteomic coverage of unpurified cell extracts [43]. Therefore, the current study extends these previous studies by examining proteomic alterations in purified cytosolic and nuclear fractions in order to globally assess sub-cellular protein distribution. Reciprocal labeling also was incorporated to identify probable contaminants which were removed from the analyses. We also extended previous protein identification by more detailed follow-up kinetic studies of some selected important proteins. This study identified and measured 2856 cytosolic proteins by 2 or more peptides at >99% confidence and 884 nuclear proteins. DAVID™ and IPA™ ontological analyses identified significantly up- and down-regulated proteins as well as significantly affected canonical pathways.

## Materials and methods

### Cells and viruses

#### Cell lines and media

Mouse L929 fibroblast cells (L929) were grown in suspension in Joklik’s modified minimal essential medium (J-MEM) (Gibco, Grand Island, NY) supplemented to contain 5% fetal bovine serum (FBS) (Invitrogen Canada Inc., Burlington, Ontario), and 2 mM L-glutamine as described [32]. Cells were sub-cultured daily.

Human HeLa cells were cultured as monolayers in Dulbecco’s modified MEM (D-MEM) supplemented with 0.2% (w/v) glucose, 10% FBS (Invitrogen), 2 mM l-glutamine, non-essential amino acids, and sodium pyruvate. Cells were sub-cultured 2 – 3 times each week.

#### Viruses

Reovirus strain Type 3 Dearing (T3D) is a laboratory stock. There are two commonly-used clones of T3D; the Cashdollar strain (T3D^C^) and the Fields strain (T3D^F^). We elected to use the T3D^F^ strain because a large number of genetic reagents, including temperature-sensitive mutants (reviewed in [44,45]) and T1L/T3D^F^ intertypic reassortants [46-48] were generated from this clone. Virus stocks were usually grown in L929 cell monolayers in J-MEM in the presence of 5% CO_2_ at 37°C as above, but with 3% FBS, 100 U/ml of penicillin, 100 μg/ml streptomycin sulfate and 100 μg/ml amphotericin-B as previously described [32].

#### Virus purification

Large quantities of reovirus T3D were grown in suspension L929 cell cultures and purified by routine procedures that make use of Vertrel-XF™ extraction and cesium chloride ultracentrifugation [49]. Purified virions were harvested and dialyzed against D-Buffer (150 mM NaCl, 15 mM MgCl_2_, 10 mM Tris, pH 7.4). Virus concentration was measured by optical density at 260 nm, using the relationship 1 ODU = 2.1 × 10^12^ particles per milliliter [34] and infectivity was titrated.

### Infections

Sets of HeLa cells were infected with gradient-purified T3D at a multiplicity of infection (MOI) of 7 PFU per cell. For routine infections not destined for SILAC analysis (i.e. for photomicrography, virus growth kinetic determinations, or Western blot analyses – see below), cells were harvested at various time points (0 – 72 hours post infection; hpi), then fractionated as described below.

For SILAC labeling, HeLa cells were adapted through 3 passages (=6 doublings) into D-MEM media provided in a SILAC™ Phosphoprotein Identification and Quantification Kit (Invitrogen Canada Inc.; Burlington, Ontario), supplemented as above (except without non-essential amino acids), and with 10% dialyzed FBS (Invitrogen), plus 100mg each of normal (**L**; ^12^C_6_-lysine and ^12^C_6_-/^14^N_4_-arginine) or “heavy” (**H**; ^13^C_6_-lysine [6.0Da difference] and ^13^C_6_-/^15^N_4_-arginine [10.0Da] difference) per liter of D-MEM. Once HeLa cells had grown through six doublings in appropriate SILAC media, sets of cells were infected with gradient-purified T3D or were mock infected with diluent. Cells were overlaid with appropriate SILAC media and cultured for 24 hours. In one experiment (Run #1) the **L** cells were infected and the **H** cells were mock infected, whereas **H** cells were infected and **L** cells were mock infected in the reciprocal labeling experiment (Run #2).

### Microscopy

HeLa cells were allowed to attach to 6-well culture dishes or onto sterile glass cover slips in 6-well culture dishes and incubated overnight at 37°C. Cells, at approximately 70% confluency, were washed twice with 1X PBS and T3D was added to each culture at an MOI of 7. Virus was adsorbed to cells on ice for one hour to ensure infection synchronization. Mock-infected cells received only diluent. Cells were overlaid with complete pre-warmed media and cells were then incubated for various periods of time from 0 to 72 hours at 37°C.

Infections were monitored at various times and aliquots taken for cell viability determinations, using trypan blue and ensuring > 200 cells were counted at each time point, and for virus titrations. Cell monolayers were also examined with a Nikon TE-2000 and cells were photographed with a Canon-A700 digital camera. Images were imported into Adobe and slight adjustments made in brightness and contrast, but which did not alter image context with respect to each other.

### Cell fractionation

Infected and mock-infected cells were harvested at various times post-infection and counted. Aliquots of each culture were saved for virus titration to verify infection status. Non-SILAC-labeled cells were individually processed. For comparative SILAC assays, equivalent numbers of **L** and **H** cells in each experimental run were confirmed to contain equivalent amounts of protein by BCA™ Protein Assay (Pierce; Rockford, IL) and were mixed together 1:1. Harvested cells were washed 3× in >50 volumes of ice-cold Phosphate Buffered Saline (PBS). Washed cells were resuspended in 250 μl of ice-cold PBS supplemented with 1.5× complete™ Protease Inhibitor (Pierce) and lysed by the addition of 1/10^th^ volume of 5% NP-40. Cells were incubated for 30 min with periodic mixing then centrifuged at 500×g for 10 min to pellet nuclei. The supernatants (cytosol and soluble membranes) were transferred to fresh microfuge tubes and the nuclear pellets were resuspended in 250 μl of PBS supplemented with 1× complete™ Protease Inhibitor + 10% sucrose + 0.44% NP-40 and nuclei re-pelleted, with the 2^nd^ supernatants added to the first ones. The nuclei were then washed 4 times with 1ml of PBS + 0.25× complete™ Protease Inhibitor + 10% sucrose. Washed nuclei were extracted by a new 2-step MS-compatible procedure [20]. Briefly, nuclei were resuspended in 150 μl of High Salt Extraction Buffer (620 mM NaCl, 1 mM DTT, 1 mM MgSO_4_, 10 mM HEPES, pH 8.0), freeze-thawed, sonicated, insoluble material pelleted at 17,000×g for 10 min, and the supernatants transferred to fresh microfuge tubes. The insoluble pellets were resuspended in 50 μl of 8 M urea, freeze-thawed, sonicated, insoluble material pelleted at 17,000×g for 10 min, and the 2^nd^ supernatants combined with the first.

Protein content in each fraction was determined by BCA Protein Assay (Pierce) and bovine serum albumin standards. The cytosolic and nuclear fractions were stored at -80°C until further processing took place.

### Immunoblotting

Equivalent cytosolic and nuclear fractions were resolved by either 10% linear mini sodium dodecyl sulfate polyacrylamide gel electrophoresis (SDS-PAGE, (8.0 × 6.5 × 0.1 cm)) at 180 V for 60 min. or in 4-16% gradient SDS-PAGE (8.0 × 16.0 × 0.1 cm) at 20 mA per gel for 5.5 hours (or overnight for a cumulative total of 110 mAhr per gel). Proteins were transferred to 0.2 μm polyvinylidene difluoride (PVDF) membranes at 20 V for 40 min with a Semi-dry apparatus (BioRad), and protein transfer was confirmed by Ponceau-S staining. The membranes were blocked with 5% (w/v) skim milk in Tris-buffered saline with Tween-20 (TBST; 50 mM Tris, 150 mM NaCl, 0.05% Tween 20, pH 7.4) and probed with various primary antibodies. Primary antibodies were: in-house produced rabbit anti-reovirus, α- GAPDH (Cell Signaling, cat#2118), α-Mx1 (Ori-Gene # TA308496), α-PARP (Cell Signaling, cat#9541), α-ISG15 (Rockland, cat#200-401-438), α-IFIT (Abcam, cat#ab55837), and α-SAMD9 (Sigma, cat#HPA021318); and mouse α-Actin (Sigma, cat#A5441) and anti-STAT1 (Cell Signaling, cat#9176). Appropriate secondary horseradish peroxidase (HRP)-conjugated rabbit anti-mouse or goat anti-rabbit (Cell Signaling, cat#7076, cat#7074, respectively) were used to detect immune complexes. Bands were developed by enhanced chemiluminescence and imaged with an Alpha Innotech FluorChemQ MultiImage III instrument.

### Comparative SILAC analyses

#### Protein digestion and peptide fractionation

After protein concentration determinations, SILAC-labeled samples were diluted with freshly made 100 mM ammonium bicarbonate to concentrations of ~1 mg/ml and pH ~8. Three hundred μl of each sample (~300 μg of protein) were reduced with dithiothreitol (DTT), alkylated with iodoacetic acid, quenched with additional DTT, and trypsin-digested overnight at 37°C with 6 μg of sequencing grade trypsin (Promega, Madison, WI) as previously described [19]. Digests were dried.

Tryptic peptides were fractionated by an orthogonal 2-dimensional reverse-phase (RP) high pH – RP low pH procedure [50,51]. Lyophilized tryptic digests were dissolved in 200 μl of 20 mM ammonium formate pH 10 (Buffer A), injected onto a 1×100 mm XTerra (Waters, Milford, MA) column and fractionated with a 0.67% acetonitrile per minute linear gradient (Agilent 1100 Series HPLC system, Agilent Technologies, Wilmington, DE) at a flow rate of 150 μL/min. Sixty 1-min fractions were collected (covering ~40% acetonitrile concentration range) and concatenated [51,52], with the last 30 fractions combined with the first 30 fractions in sequential order (i.e. #1 with #31; #2 with #32, etc.). Combined fractions were vacuum-dried and re-dissolved in buffer A for the second dimension RP separation (0.1% formic acid in water).

A splitless nano-flow Tempo LC system (Eksigent, Dublin, CA) with 20 μL sample injection via a 300 μm×5 mm PepMap100 pre-column (Dionex, Sunnyvale, CA) and a 100 μm×200 mm analytical column packed with 5 μm Luna C18(2) (Phenomenex, Torrance, CA) were used in the second dimension separation prior to MS analysis. Both eluents A (water) and B (acetonitrile) contained 0.1% formic acid as an ion-pairing modifier. A 0.33% acetonitrile per minute linear gradient (0-30% B) was used for peptide elution, providing a total 2-hour run time for each of the 30 concatenated samples.

#### Mass spectrometry, bioinformatics, and data mining

A QStar Elite mass spectrometer (Applied Biosystems, Foster City, CA) was used in a data-dependent MS/MS acquisition mode. One-second survey MS spectra were collected (m/z 400-1500) followed by MS/MS measurements on the 3 most intense parent ions (80 counts/sec threshold, +2 - +4 charge state, m/z 100-1500 mass range for MS/MS), using the manufacturer’s “smart exit” (spectral quality 5) settings. Previously targeted parent ions were excluded from repetitive MS/MS acquisition for 60 sec (50 mDa mass tolerance) and the bias correction option was used to correct for small pipetting errors. Raw data files (30 for each of the 4 experimental run samples) were submitted for simultaneous search using standard SILAC settings for QStar instruments and were analyzed by Protein Pilot®, version 4.0, using the non-redundant human gene database (NCBInr, released March 2011, downloaded from http://ftp.ncbi.nih.govrefseqH_sapiensmRNA_Prot, containing 37,391 entries). Proteins, their confidences, and their expression ratios, expressed as infected: mock (I:M), were returned with gi accession numbers. Only proteins for which at least 2 fully trypsin digested **L** and **H** peptides were detected at >99% confidence were used for subsequent comparative quantitative analyses. The false discovery rate (FDR), defined as the percentage of reverse proteins identified against the total protein identification, was determined to be < 0.8%.

Each of the 4 datasets were normalized, essentially as described [53] to allow dataset merging and comparison. Briefly, every I:M ratio was converted into log space to determine geometric means and standard deviations in each dataset. Every protein’s log_2_ I:M ratio was then converted into a z-score, using the formula:

$$Z-\text{score}\left(\sigma \right)\;\mathrm{of}\;\left[b\right]=\frac{\begin{array}{c} {\mathrm{Log}}_{2}I:M\left[b\right]–\mathrm{Average}\;\mathrm{of}\\ \;\left(\log_{2}\;\text{of}\;\text{each}\;\text{member},a\ldots .n\right) \end{array}}{\begin{array}{c} \text{Standard}\;\text{deviation}\;\text{of}\\ \;\left(\log_{2}\;\text{of}\;\text{each}\;\text{member},a\ldots .n\right) \end{array}}$$

where “b” represents an individual protein in a dataset population a….n, and z-score is the measure of how many standard deviation units (expressed as “σ”) that protein’s log_2_ I:M ratio is away from its population mean. Thus, a protein with a z-score > 1.960σ indicates that protein’s differential expression lies outside the 95% confidence level, > 2.576σ indicates outside the 99% confidence level, and 3.291σ indicates 99.9% confidence. Z-scores >1.960 were considered significant. Proteins that obtained significant positive z-scores in one labeling experiment, but that also obtained significant negative z-scores in the reciprocal labeling experiment were assumed to be contaminants, were removed from analysis, and z-scores iteratively re-calculated. Gi numbers of all significantly regulated proteins were converted into HGNC identifiers by Uniprot (http://www.uniprot.org/) and HGNC terms were submitted to and analyzed by STRING [54,55] and by the DAVID bioinformatic suite at the NIAID, version 6.7 [56,57] and gene ontologies examined with the “FAT” datasets. The gi numbers were also submitted to, and pathways constructed with, Ingenuity Pathway Analysis software (IPA™).

## Results and discussion

### T3D replicates in HeLa cells

**Figure 1:**
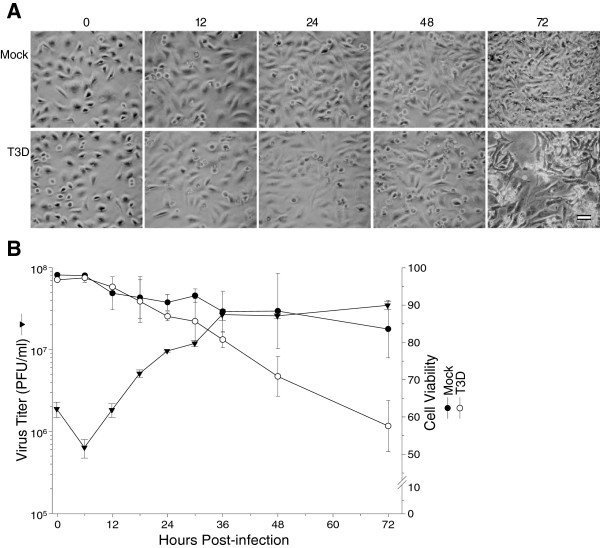
**Confirmation of HeLa cell infectivity. A**, HeLa cells were mock-infected (top), or infected with MRV strain T3D at an MOI of 7 (bottom). Cells were imaged at indicated times post-infection (labeled at top) with a Nikon TE-2000 inverted microscope and photographed with a Canon A700 digital camera. Images were imported into Adobe and slight adjustments made in brightness and contrast, that did not alter image context. Scale bar is 50 μm. **B**, Kinetics of virus production (▼) and cell viability after HeLa cells were infected (○) or mock-infected (●) with T3D. Error bars represent standard error of the mean from 2 or more replicates.

Reoviruses are routinely cultured and titrated in mouse L929 cells [22,23]. The virus also is capable of growing in HeLa cells [35]. We evaluated the time course and confirmed the capacity of T3D to enter and replicate in HeLa cells, and to cause cytopathic effect (CPE), under various infection conditions (Figure 1). Phase-contrast microscopy indicated mock-infected cells showed no evidence of virus infection and CPE was not noticeable until almost a week later, presumably due to nutrient deprivation. T3D-infected HeLa cells also showed little, if any, noticeable CPE until approximately 72 hpi (Figure 1A). Differences in CPE were detectable by trypan blue exclusion, with mock-infected cells showing gradual loss of cell viability from 0 to 72 hpi (Figure 1B). Differences in trypan blue-measured cell viability between the mock- and T3D-infected samples began to manifest at 24 hpi and became more apparent later (Figure 1B). Virus titers initially declined, corresponding to virus uncoating, and began to increase after 6 hpi, was exponential from 6 to 36 hpi, and plateaued after this point. Thus, we elected to use an initial MOI of 7, the same amount of virus used in our earlier quantitative proteomic analyses [19-21], and to examine comparative quantitative differences at the 24 hpi time point, prior to significant CPE development, to allow more meaningful comparison to these other studies.

### Identification of altered host proteins

**Figure 2:**
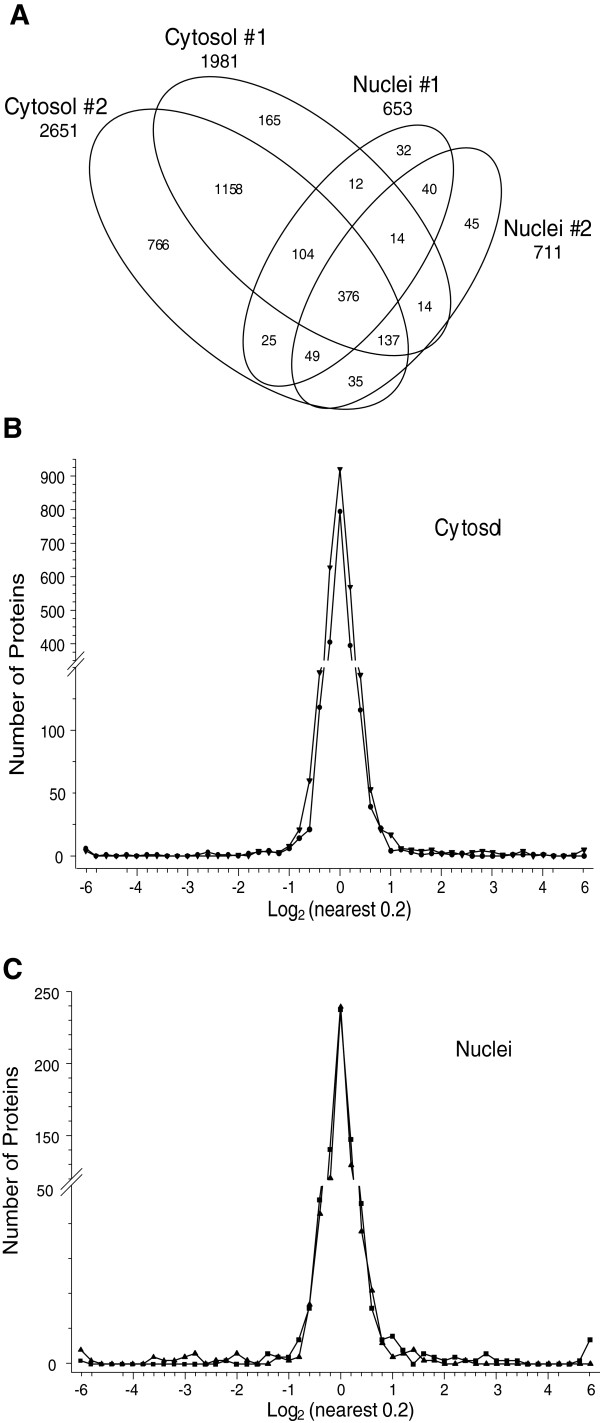
**Distributions of proteins identified in various experiments. A**, Venn diagram of numbers of identified proteins from various analyses. **B**, Frequency distributions of identified proteins in both cytosolic sample sets, with I:M ratios expressed as Log_2_ values. Positive values represent up-regulated host proteins in virus-infected cells; negative values represent down-regulated host proteins. **C**, Frequency distributions of identified proteins in both nuclear sample sets. Note that distributions are not identical, with different numbers of proteins differentially expressed away from the major central peak. Characteristics of all peptide and protein distributions, mean log_2_ I:M ratios and standard deviations of log_2_ I:M ratios are shown in Table 1.

HeLa cells were infected (or mock) in duplicate and purified cytosolic and nuclear fractions obtained. Viruses generally induce profound alterations in infected cells. To rule out the possibility that infection might lead to profound alterations, including significant nuclear leakage by 24 hpi, which could affect our interpretations of protein localization, we determined the distribution of annotated nuclear proteins within each of our **L**-labeled cytoplasmic fractions (Additional file 1: Figure S1). 922 of the 2137 (43.1%) **L**-labeled proteins in the mock-infected cytoplasmic sample had GO nuclear annotations, whereas 43.6% of the 1583 **L**-labeled proteins in the infected cytoplasmic sample had GO nuclear annotations, suggesting no significant nuclear leakage by 24 hpi in the infected samples. 2D-HPLC/MS processing of the mixed samples identified 73,969 **H:L** peptide pairs, corresponding to 3230 proteins, at ≥99% confidence. 2067 proteins were found in the cytosolic fraction in experimental run #1 and 2787 were present in the run #2 cytosolic fraction (Table 1). Exclusion of those proteins identified by only a single peptide, as well as those that probably represent contaminants (see next paragraph), further reduced the number of identified proteins to 1981 in the run #1 cytosolic fraction (Table 1; Figure 2A). Using similar criteria, we analyzed 2651 proteins in the reciprocally-labeled cytosolic fraction (run #2) and 653 and 711 nuclear proteins (Table 1; Figure 2A).

**Table 1:** **Number of peptides, proteins, log**_**2 **_**Infected: Mock (I:M) ratio means and standard deviation, and Z-scores of SILAC-measured HeLa cell proteins after T3D infection**

	**Cytosol**		**Nuclei**	
	**Run #1**	**Run #2**	**Run #1**	**Run #2**
Total number of peptide pairs^1^	22,591	34,393	8,011	8,974
Total number of proteins^2^	2,067	2,787	670	728
Number of proteins analysed^3^	1,987	2,684	635	686
Mean Log_2_ I:M ratios	0.0147	0.0126	0.0004	0.0187
Standard deviation of Log_2_ I:M ratios	0.3382	0.4211	0.4664	0.3423
Number of proteins at Z-score cutoff of: ± 1.960σ (95%)	33, 22^4^	50, 20	7, 4	20, 12
± 2.576σ (99%)	24, 11	29, 11	5, 2	14, 8
± 3.291σ (99.9%)	14, 7	23, 8	2, 2	8, 4

^1^ Total number of H:L peptide pairs for all proteins identified at confidence level ≥ 99%.

^2^ Total number of proteins identified at confidence level ≥ 99%.

^3^ Number of proteins analyzed after those identified by only a single peptide, as well as possible contaminants, removed.

^4^ First value is number of up-regulated proteins outside the indicated confidence level; second number is number of down-regulated proteins outside the indicated confidence level.

Each protein’s infected-to-mock (I:M) **H:L** or **L:H** ratio was converted into a z-score to normalize the data and facilitate comparisons of each dataset as described in Materials and Methods (and in [19]). A number of proteins were found to have significantly high or low log_2_ values and corresponding z-scores in either the **H:L** or **L:H** labeling scheme, but z-scores that were significantly regulated in the opposite direction in the reciprocal labeling experiment. These included hornerin, keratins, some S100 calcium-binding proteins, and some other proteins (Additional file 2: Table S1; rows 3111-3243). These significant reciprocal values could arise if, for example, exogenous unlabeled (= **L**-labeled) proteins were introduced during processing. Thus, such proteins were assumed to represent possible contaminants and were removed from further analysis. Z-scores were iteratively recalculated until all probable contaminants had been removed.

Stratification of each protein’s I:M ratio and its corresponding z-score indicated that numerous proteins in each sample could be considered significantly regulated. For example, of the 1987 proteins identified in the cytosolic run #1 preparation, 33 were up-regulated at 95% confidence and 14 were also up-regulated at 99.9% confidence (Table 1). Twenty-two proteins in the same dataset were down-regulated at 95% confidence, and seven of these proteins were also down-regulated at 99.9% confidence. Inspection of protein I:M ratios and z-scores indicated that most proteins differentially regulated at >95% confidence had I:M ratios altered by > 1.6-fold. Thus, for proteins observed multiple times, we applied several filtering levels. We considered them significantly regulated if at least one of their observations had a z-score ≥ 1.960σ, if the other observation in the same type of fraction (cytosolic or nuclear) was no more than 0.75σ in the opposite direction, if most of the peptides used to determine the protein regulation were differentially regulated at >95% confidence, if peptide regulation variability, as measured by standard error of the mean, was relatively small, and if the average I:M ratio was > 1.6-fold. Application of these filters resulted in removal of several proteins which might otherwise have been considered significantly regulated. For example, mitochondria-associated granulocyte macrophage CSF signaling molecule (gi|27363461) had an I:M ratio of 1.63 (Additional file 2: Table S1; row 3133) but was excluded because two peptides had measured I:M ratios of ~1.22 and one peptide had a measured ratio of 100 (STD = 12.64). For proteins observed a single time, they were considered significantly regulated if the z-score was ≥ 1.960σ and if the I:M ratios were altered > 1.6-fold. Using the above criteria, we identified and measured 45 proteins that were significantly up-regulated and 19 proteins that were significantly down-regulated in the cytosolic fraction and 16 proteins that were significantly up-regulated and 9 proteins that were significantly down-regulated in the nuclear fraction (Table 2).

**Table 2:** Significantly-regulated HeLa cell proteins after T3D infection

						**Run # 1 (H:L)**			**Run # 2 (L:H)**		
	**Accession**	**HGID**	**Name**	**Inf / Mock**	**(±S.E.M.)**	**# Pep**	**% Cov**	**Z-Score**	**# Pep**	**% Cov**	**Z-score**
***Cytoplasmic***											
*Up-regulated*											
*Measured more than once*											
	gi|222136619	MX1	myxovirus resistance protein 1	9.76	0.52	8	55.9	10.925	23	51.7	7.427
	gi|94536771	CCD56	coiled-coil domain containing 56	5.48	1.62	5	56.6	0.223	3	20.7	15.154
	gi|27881482	DDX58	DEAD/H (Asp-Glu-Ala-Asp/His) box polypeptide RIG-I	4.76	0.83	3	27.7	7.191	12	23.2	5.203
	gi|72534658	IFIT3	interferon-induced protein with tetratricopeptide repeats 3	4.65	0.39	4	25.3	7.260	14	46.9	5.067
	gi|4826774	ISG15	ISG15 ubiquitin-like modifier	4.60	0.51	14	47.3	6.164	14	53.9	5.442
	gi|116534937	IFIT1	interferon-induced protein with tetratricopeptide repeats 1 isoform 2	3.80	1.36	2	40.4	11.782	8	25.1	3.308
	gi|156105693	PR285	PPAR-alpha interacting complex protein 285 isoform 1	3.45	1.53	2	22.0	5.564	5	10.6	4.109
	gi|153082755	IFIT2	interferon-induced protein with tetratricopeptide repeats 2	3.37	0.81	3	33.7	1.291	11	33.1	4.978
	gi|6274552	STAT1	signal transducer and activator of transcription 1 isoform alpha	3.23	0.39	13	44.4	4.386	34	50.4	4.170
	gi|45007007	OAS3	2'-5'oligoadenylate synthetase 3	3.00	0.32	4	19.5	4.560	9	17.3	3.761
	gi|208973246	DHPR	quinoid dihydropteridine reductase	2.40	0.48	2	27.9	0.693	4	20.5	4.171
	gi|38201706	SAMD9	sterile alpha motif domain containing 9	2.15	0.48	1	18.8	2.560	10	15.4	2.648
	gi|4506103	E2AK2	eukaryotic translation initiation factor 2-alpha kinase 2	1.97	0.72	3	45.7	2.503	10	16.2	2.374
	gi|24307901	IFI35	interferon-induced protein 35	1.92	0.44	3	29.2	2.119	5	18.4	2.508
	gi|166706903	GBP1	guanylate binding protein 1, interferon-inducible, 67 kDa	1.85	0.88	5	25.7	1.682	11	27.2	2.393
	gi|5031863	LG3BP	galectin 3 binding protein	1.81	0.86	6	19.5	1.731	12	29.2	2.303
	gi|22035653	APOL2	apolipoprotein L2	1.80	0.53	4	30.3	1.501	6	32.6	2.507
	gi|27477136	ZCCHV	zinc finger antiviral protein isoform 1	1.80	0.69	2	26.2	2.245	16	25.5	2.007
	gi|20270303	MIRO2	ras homolog gene family, member T2	1.79	0.92	2	15.7	9.955	6	15.7	−0.055
	gi|4757876	BST2	bone marrow stromal cell antigen 2	1.68	0.38	5	19.4	1.610	5	18.3	2.201
	gi|188528628	PNPT1	polyribonucleotide nucleotidyltransferase 1	1.63	0.20	13	47.6	2.221	24	36.8	1.575
*Measured once*											
	gi|156415992	ATP8*	transcription factor B1, mitochondrial	84.09	0.71				2	9.2	15.154
	gi|4557321	APOA1	apolipoprotein A-I preproprotein	7.99	0.97				2	23.2	7.089
	gi|11342664	MX2	myxovirus resistance protein 2	7.72	0.57				6	11.3	6.972
	gi|4502511	CO9	complement component 9 precursor	7.26	4.88				2	5.0	6.760
	gi|166706909	IF44L	interferon-induced protein 44-like	6.18	3.89				3	15.9	6.211
	gi|74229015	OAS1	2',5'-oligoadenylate synthetase 1 isoform 3	5.85	0.86				3	11.6	6.024
	gi|19923717	DTX3L	deltex 3-like	3.68	3.31				4	9.7	4.438
	gi|74229019	OAS2	2'-5'-oligoadenylate synthetase 2 isoform 1	3.50	2.14				2	4.9	4.263
	gi|41350201	EPN1	epsin 1 isoform c	3.20	2.53				2	5.5	3.953
	gi|5174751	YAP1	Yes-associated protein 1, 65 kD	3.12	1.32				3	7.9	3.867
	gi|222831595	DDX60	DEAD (Asp-Glu-Ala-Asp) box polypeptide 60	2.92	1.56				5	7.6	3.636
	gi|15208660	RO52	tripartite motif protein 21	2.69	0.63				8	22.7	3.364
	gi|110825982	HERC5	hect domain and RLD 5	2.50	0.86				2	4.7	3.104
	gi|4557499	CTBP2	C-terminal binding protein 2 isoform 1	2.49	1.36				7	25.8	3.093
	gi|4506191	PSMB10	proteasome beta 10 subunit proprotein	2.21	0.71				2	7.3	2.688
	gi|73747915	TAP2	transporter 2, ATP-binding cassette, sub-family B isoform 1	2.16	0.70				3	10.8	2.615
	gi|38016914	SAMH1	SAM domain- and HD domain-containing protein 1	2.16	0.48				6	21.7	2.610
	gi|126012562	LRP1	low density lipoprotein-related protein 1	2.11	0.60				3	2.9	2.529
	gi|4503445	TYPH	endothelial cell growth factor 1 (platelet-derived) precursor	2.10	0.85				2	18.1	2.504
	gi|6912630	IFIT5	interferon-induced protein with tetratricopeptide repeats 5	2.05	0.63				3	13.9	2.435
	gi|112789562	IF16	interferon, gamma-inducible protein 16	1.95	0.64				6	21.8	2.263
	gi|45580709	UN93B	unc-93 homolog B1	1.93	0.77				2	4.2	2.231
	gi|9665248	TAP1	transporter 1, ATP-binding cassette, sub-family B	1.88	0.78				2	11.8	2.135
	gi|52630342	HLA-C	major histocompatibility complex, class I, C precursor	1.80	0.34				20	45.4	1.982
*Down-regulated*											
*Measured more than once*											
	gi|134288865	S4A7	solute carrier family 4, sodium bicarbonate cotransporter, member 7	0.064	0.21	2	21.7	−3.832	2	3.8	−15.807
	gi|29294627	PPFIBP1	PTPRF interacting protein binding protein 1 isoform 1	0.30	0.21	3	26.5	−10.860	3	11.0	0.328
	gi|4506431	RASA1	RAS p21 protein activator 1 isoform 1	0.36	0.06	1	16.5	−1.418	2	1.8	−4.688
	gi|5453704	PRAF3	ADP-ribosylation-like factor 6 interacting protein 5	0.56	0.18	4	40.4	−3.941	2	29.8	0.235
	gi|21956645	MTPN	myotrophin	0.56	0.08	9	49.1	−4.823	8	39.0	0.091
*Measured once*											
	gi|167466177	CDC27	cell division cycle protein 27 isoform 1	0.010	0.36				2	4.0	−15.807
	gi|8922549	ASF1B	anti-silencing function 1B	0.33	0.000				2	23.8	−3.834
	gi|33598948	PLCG1	phospholipase C gamma 1 isoform a	0.33	0.34				2	6.9	−3.803
	gi|46049063	XPO6	exportin 6	0.34	0.05				2	5.2	−3.706
	gi|116734706	I2BP2	interferon regulatory factor 2 binding protein 2 isoform B	0.36	0.38				3	13.7	−3.517
	gi|116686114	FERM1	kindlin-1	0.38	0.004				3	12.3	−3.348
	gi|5453543	AK1C1	aldo-keto reductase family 1, member C1	0.39	0.05	14	72.8	−4.099			
	gi|63025188	HERC4	hect domain and RLD 4 isoform a	0.41	0.000				2	2.5	−3.077
	gi|14589951	RPAB1	DNA directed RNA polymerase II polypeptide E	0.46	0.14				6	21.4	−2.683
	gi|51317370	NDUA6	NADH dehydrogenase (ubiquinone) 1 alpha subcomplex, 6, 14 kDa	0.47	0.000				2	14.9	−2.637
	gi|10190746	RDH14	retinol dehydrogenase 14 (all-trans and 9-cis)	0.51	0.38				2	8.6	−2.306
	gi|13236559	MMTA2	hypothetical protein LOC79169	0.54	0.01				2	35.0	−2.138
	gi|115430235	UHRF1	ubiquitin-like with PHD and ring finger domains 1 isoform 1	0.55	0.10				2	11.1	−2.105
	gi|71773010	AP1G1	adaptor-related protein complex 1, gamma 1 subunit isoform a	0.55	0.25	4	22.3	−2.570			
***Nuclear***											
*Up-regulated*											
*Measured more than once*											
	gi|4826774	ISG15	ISG15 ubiquitin-like modifier	4.05	0.40	5	42.4	4.065	4	23.0	6.275
	gi|19923236	SP100	nuclear antigen Sp100 isoform 2	2.96	0.93	4	18.1	2.812	8	26.2	4.880
	gi|112789562	IF16	interferon, gamma-inducible protein 16	2.92	0.40	2	11.8	2.384	6	19.3	4.879
	gi|67089149	PML	promyelocytic leukemia protein isoform 1	2.34	0.60	2	8.4	2.758	9	21.7	3.496
	gi|4757876	BST2	bone marrow stromal cell antigen 2	2.03	0.56	3	18.3	2.512	2	13.9	2.290
*Measured once*											
	gi|4503253	DAD1	defender against cell death 1	5.22	0.62	3	17.7	5.110			
	gi|188219599	NMI	N-myc and STAT interactor	4.33	1.17				2	16.9	6.125
	gi|24307901	IFI35	interferon-induced protein 35	3.60	0.93				3	15.6	5.347
	gi|4758024	COIL	coilin	2.46	0.83	2	12.8	2.788			
	gi|5453690	DNJB1	DnaJ (Hsp40) homolog, subfamily B, member 1	2.32	0.000				2	11.8	3.499
	gi|58530840	DESP	desmoplakin isoform I	2.09	0.91				3	9.3	3.054
	gi|156104878	GLSK	glutaminase	2.03	0.75				3	4.8	2.931
	gi|27477136	ZCCHV	zinc finger antiviral protein isoform 1	1.95	0.74				2	2.2	2.754
	gi|4507951	1433F	tyrosine 3-monooxygenase/tryptophan 5-monooxygenase activation protein, eta polypeptide	1.89	1.06				2	22.8	2.633
	gi|5901998	LSM6	Sm protein F	1.85	0.60				3	56.3	2.538
	gi|4506003	PP1A	protein phosphatase 1, catalytic subunit, alpha isoform 1	1.70	0.95				4	13.9	2.184
*Down-regulated*											
*Measured more than once*											
	gi|55953087	NOG1	G protein-binding protein CRFG	0.10	0.21	2	4.4	−13.385	2	6.3	−1.353
	gi|4503453	EDF1	endothelial differentiation-related factor 1 isoform alpha	0.63	0.17	4	28.4	−0.672	4	41.2	−2.991
	gi|4507555	LAP2A	thymopoietin isoform alpha	0.64	0.03	10	15.3	−2.464	10	12.5	−0.479
*Measured once*											
	gi|88853069	VTNC	vitronectin precursor	0.013	0.04	2	4.8	−13.385			
	gi|25777615	PSD7	proteasome 26S non-ATPase subunit 7	0.39	0.30				3	12.3	−4.046
	gi|4757732	AIFM1	programmed cell death 8 isoform 1	0.40	0.18				2	18.4	−3.870
	gi|57863257	TCPA	T-complex protein 1 isoform a	0.50	0.04				2	5.2	−2.951
	gi|4506701	RS23	ribosomal protein S23	0.54	0.19				4	32.9	−2.647
	gi|151108473	FIS1	tetratricopeptide repeat domain 11	0.63	0.000				2	7.2	−1.990

*: Obsolete record; removed from NCBI database.

### HeLa cell proteins up-regulated by T3D infection are associated with defense responses, immune responses, macromolecular binding, regulation of immune effector processes, and responses to virus

**Figure 3:**
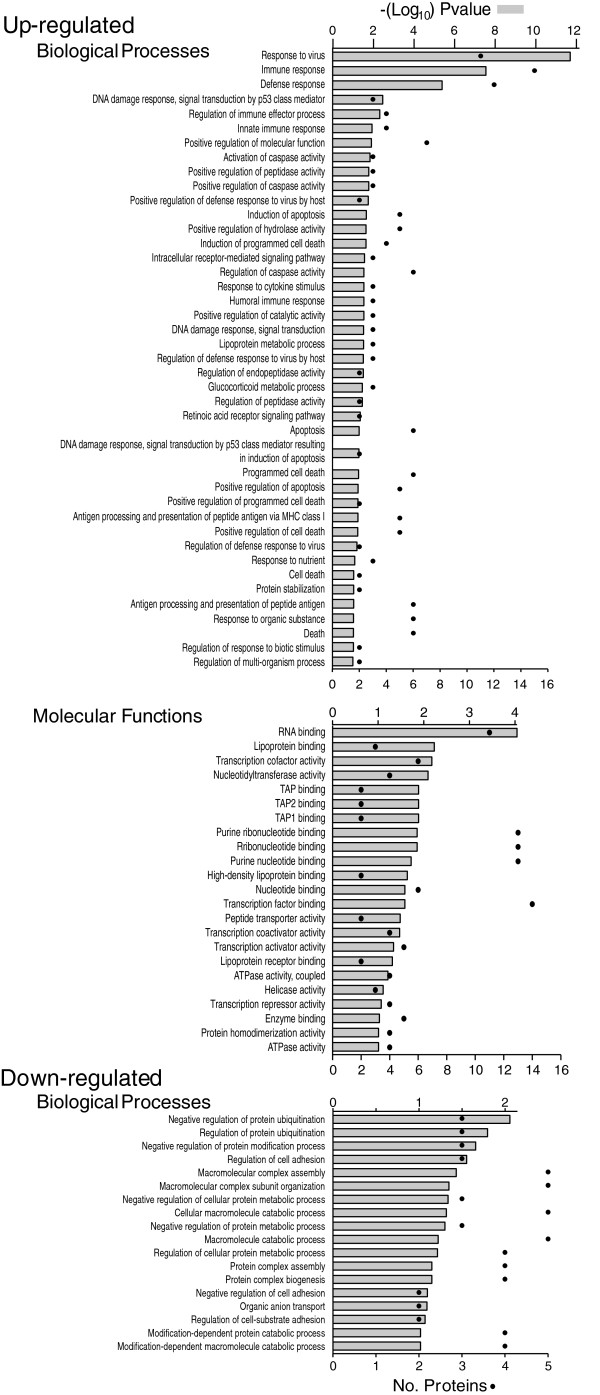
**Gene ontology analyses of up-regulated and down-regulated proteins.** Sets of up- and down-regulated proteins identified in Table 2 were separately imported into the DAVID gene ontology suite of programs at the NIAID, gene identifications converted by that program, and ontological functions determined by GOTERM.

Proteins, and their levels of regulation, were analyzed several ways. Protein gi numbers were imported into Uniprot (http://www.uniprot.org/) and converted into HUGO nomenclature committee (HGNC) identifiers. Significantly up-regulated and down-regulated proteins (95% confidence interval) were then imported into DAVID [56,58] and gene ontologies identified (Figure 3).

Up-regulated proteins were assigned to 42 GOTERM biological processes at 95% confidence (Figure 3, upper), that included responses to viruses, immune responses, defense responses, regulation of immune effector processes and numerous related processes. Up-regulated proteins were also assigned to 23 functional groups (Figure 3, middle) including primarily macromolecular binding and transcription cofactor and nucleotidyltransferase activities.

**Figure 4:**
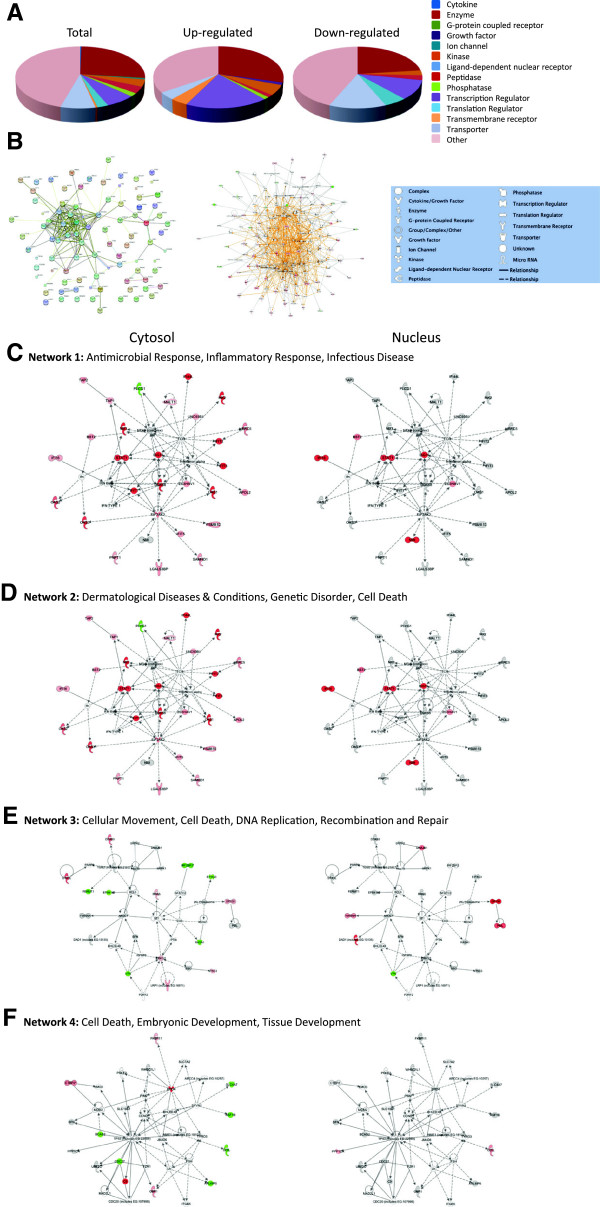
**Molecular pathways of regulated proteins.** Proteins and their levels of regulation were imported into the Ingenuity Pathways Analysis (IPA™) tool and interacting pathways were constructed. **A**, Ontological classifications of all measured proteins (Total) as well as those significantly up- and down-regulated. **B**, Merged networks, determined by uploading the regulated proteins only into STRING [54,55] (left), or by uploading all molecules and their degree of regulation into IPA™ (middle). **C-F**, The top 4 IPA networks, identified at 95% confidence and each of which contained 12 or more “focus” molecules (molecules significantly up- or down-regulated), with pathway names indicated. Solid lines: direct known interactions; dashed lines: suspected or indirect interactions. Significantly regulated proteins identified in either the cytosolic or nuclear fractions were overlaid onto each network; red: significantly up-regulated proteins; pink: moderately up-regulated proteins; grey: proteins identified but not significantly regulated; light green: moderately down-regulated proteins; dark green: significantly down-regulated proteins; white: proteins known to be in network, but not identified in our study.

Protein HGNC identifiers were also uploaded into STRING [54,55] and interactions depicted (Figure 4B, left). Many immunomodulatory proteins (ie. MX1, MX2, ISG15, various IFITs and various OAS molecules) form a major interacting cluster and interact either directly or indirectly with numerous other regulated proteins.

**Figure 5:**
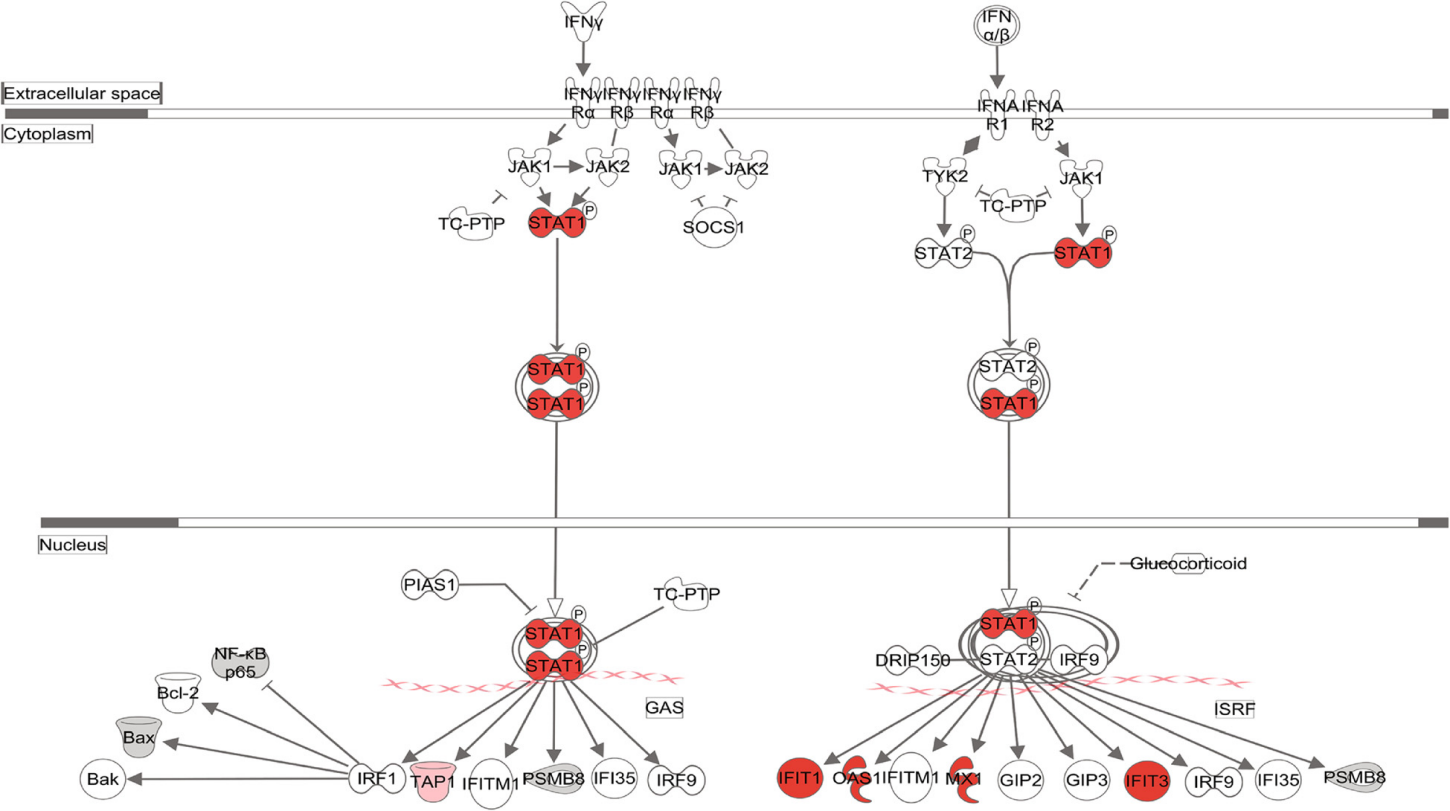
**Significantly affected canonical pathway “Interferon signaling”, as determined by IPA**^**™ **^**analysis.** Red indicates highly up-regulated proteins, light green indicates down-regulated, grey represents not significantly affected, and white represents molecules known to be part of the pathway but not identified by SILAC.

Protein gi numbers and levels of regulation were also imported into the Ingenuity Pathways Analysis (IPA™) tool to build interacting pathways. IPA indicated up-regulated proteins were enriched in cytokines, growth factors, transcription regulators and transmembrane receptors. IPA identified 8 interacting pathways at a confidence level of 95% or greater. Four of these pathways, each with 12 or more “focus” members (significantly up- or down-regulated proteins), shared common members and it was possible to build a single, merged pathway (Figure 4B, middle). Other pathways contained 2 or fewer focus molecules. The four major pathways were those involved in antimicrobial response, inflammatory response, infectious diseases, dermatological diseases and conditions, cell-to-cell signaling and interaction, cell cycle, and cell death. Regulated proteins identified in each of the purified cytosolic and nuclear fractions were overlaid onto each network (Figure 4C–F). Proteins identified in our analyses as up-regulated and present in the pathways are depicted in shades of red. Some proteins, such as IFI16, ISG15, SP100, and STAT1, were highly regulated in both the cytosol and the nucleus, whereas some, such as IFIT1, Mx1, and TRIM21, were up-regulated in one fraction but not the other. Many of the identified regulated proteins also are involved in several known canonical pathways, including interferon signaling (Figure 5), glycogen degradation, pattern recognition receptors, PDGF signaling, antigen presentation, protein ubiquitination, and others (Additional file 3: Figure S2). Many of the highly up-regulated proteins, such as STAT, ISG15 and Mx1 also represent interaction nodes within various pathways, interacting with numerous other proteins. Many of these proteins play important roles in innate immunity. In addition, the Mx proteins, which are large GTPase dynamin-like interferon-induced molecules, are important anti-viral proteins, particularly against RNA viruses [59,60]. These have been identified as up-regulated by various virus infections, including by influenza [4,7,61] and by reoviruses [62-64].

**Figure 6:**
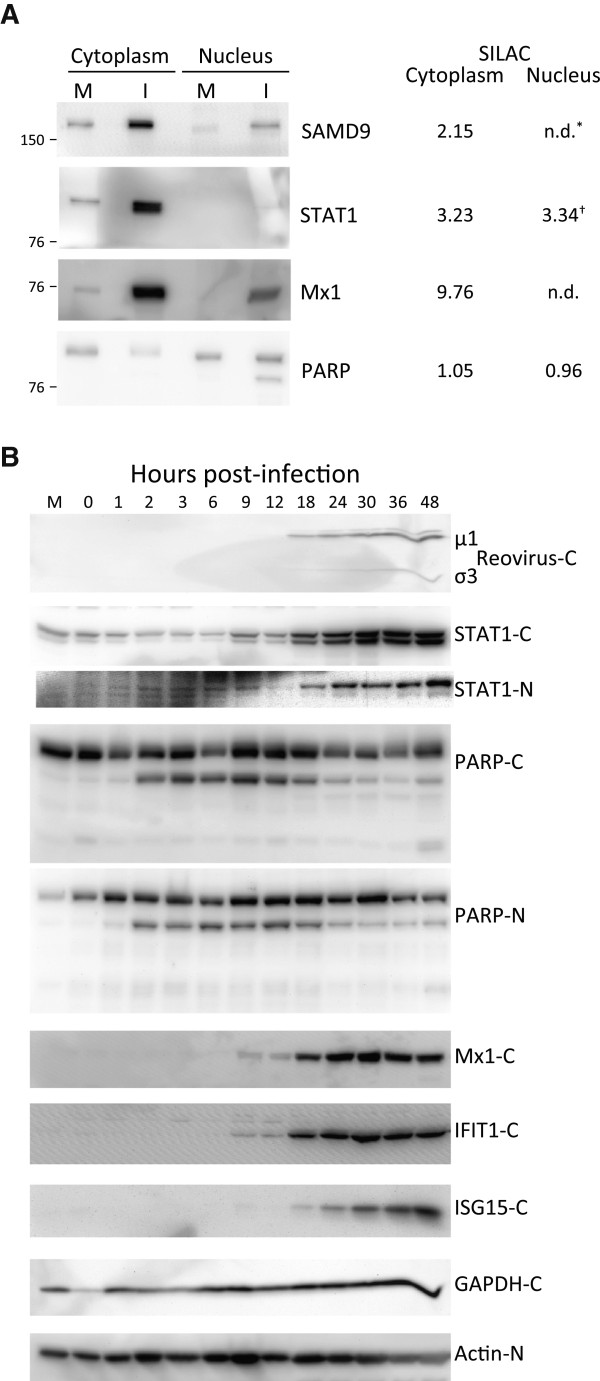
**Western blot validation of host protein regulation. A**, HeLa cells were mock-infected or infected for 24 h, or **B**, for indicated periods of time, harvested and lysed with 0.5% NP-40 detergent. The cytosolic and nuclear fractions were separately purified, dissolved in SDS electrophoresis sample buffer, and proteins resolved in 10% (**A**), or in 4-16% gradient (**B**) SDS-PAGE, transferred to PVDF, and probed with indicated antibodies. Antibody binding was detected with HRP-conjugated secondary antibodies and ECL, and visualized with an Alpha Innotech FluorChemQ MultiImage III instrument. Molecular weight standards are indicated at left and SILAC-measured ratios are indicated on right in **A**. *: not detected in indicated fraction; †: based on single peptide only.

To both validate some of the SILAC data, as well as determine temporal expression of various immune-modulated proteins, we harvested T3D-infected HeLa cells at various times, extracted the cytosolic and nuclear fractions, and immuno-probed for various molecules (Figure 6). Most Western blot results confirmed the SILAC-determined results (Figure 6A). For example, STAT1 was measured as up-regulated in both the cytoplasmic as well as nuclear fractions by SILAC and Western blot of both fractions confirmed these results. A few differences in the specific measured ratios (for example, SILAC indicated STAT1 was up-regulated 3.2–fold in the cytoplasm, but Western blot densitometry suggested the increase was approximately 5–fold) probably reflect different levels of sensitivity of the two assays. In addition, SILAC suggested the relative levels of PARP were essentially unchanged, although Western blot suggested a small decrease in cytoplasmic levels and nuclear fractions demonstrated PARP cleavage (Figure 6A). Kinetic analyses revealed more details. Reovirus proteins were not detected at early time points but became detectable by 18 hpi. STAT-1, an important effector protein that plays a key role in numerous canonical pathways (Figure 5; Additional file 3: Figure S2), and that was found up-regulated in both the cytoplasmic as well as nuclear fractions by SILAC (Figure 6A; Table 2), was detected in mock-infected cells as well as in infected cells. STAT-1 expression appeared to remain constant until 9 hpi, and then increased from 9 hpi onward, reaching levels about 3-fold higher at 24 hpi in the cytoplasm compared to mock and about 6-fold higher at later time points. Significant STAT1 increases were seen at later times, 18 hpi, in the nucleus (Figure 6B). Similarly, expression of Mx1 was detected in mock and at early times in infected cells and began to increase at 9 hpi, reaching ~8-fold higher levels by 24 hpi (Figure 6B). Mx1, IFIT1, and ISG15 were detected in cytoplasmic fractions and showed clear up-regulation by 18 hpi (by 9 hpi in the case of Mx1 and IFIT1) (Figure 6B), but none of these three proteins were detected in nuclear fractions at any time point up to 48 hpi (data not shown). PARP, cleavage of which serves as an apoptosis marker, demonstrated increased cleavage in both the cytoplasm and the nucleus as early as 2 hpi and this cleavage appeared to wane by about 18-24 hpi, shortly after reovirus proteins became detectable and shortly after the other discussed proteins started to increase, consistent with known interactions between these and other innate immune and inflammatory response molecules [65-67].

### HeLa cell proteins down-regulated by T3D infection are associated with cell death, macromolecular catabolic processes, and tissue development

DAVID assigned down-regulated proteins to 18 biological processes at 95% confidence (Figure 3, lower), that included ubiquitination regulation, cell adhesion regulation, macromolecular complex assembly and catabolic processes. IPA indicated down-regulated proteins were enriched in translation regulation and transporter factors (Figure 4A). Many down-regulated proteins also are related to mitochondrial dysfunction (Additional file 3: Figure S2). Many other proteins, such as EIF4G3, FERMT1, CDC27 and COIL were mapped to the cytoplasmic fractions (Figure 4E, F).

An apparent down-regulation in protein levels in either of the sub-cellular fractions examined could represent reduced protein biosynthesis, protein degradation, redistribution of proteins from one fraction to another, or various combinations of the above. Protein redistribution could be manifested by the same protein apparently being up-regulated in one compartment at the same time it appeared down-regulated in the other compartment. While there were numerous proteins that were detected as either up-regulated or down-regulated in one compartment and unaffected in the other compartment (i.e. IFIT3, MX2, PML, RASA1, XPO6) there were very few proteins detected that were up-regulated in one compartment and simultaneously down-regulated in the other. For example coilin (COIL) was found up-regulated about 2.5-fold in purified nuclear fractions, based upon 2 peptides; however, it appeared down-regulated by about 2-fold, but only based upon a single peptide (Additional file 2: Table S1). This molecule is an integral component of nuclear suborganelles called Cajal bodies that play roles in small RNA post-transcriptional modifications [68,69]. More detailed analyses of fates of various proteins, with regards to the possible altered biosynthesis, degradation and redistribution as a result of virus infection, are warranted.

### Similarities and differences between HeLa cell and HEK293 cell protein responses to reovirus infection

Transcriptomic responses to T3D infection have been previously reported [40]. That study identified more than 100 interferon- and NF-κB-responsive genes that were either positively or negatively regulated by T3D infection. We identified and measured 30 of these genes’ proteins and assessed correlation between the mRNA and protein levels. There was good correlation for some genes and proteins. For example, OAS1 and PML mRNA and protein levels were highly up-regulated, 9 other proteins we identified as up-regulated also had up-regulated mRNA levels, although the degree of up-regulation differed, and some proteins and genes (i.e. UBC and POLD1) were similarly non-regulated or only slightly down-regulated. Analysis of the 30 proteins after assigning proteins and genes to highly-up, slightly-up, non-regulated, slightly-down and highly-down regulated classes resulted in an r^2^ correlation of 0.62 (data not shown), slightly higher than what has been found in other studies that correlate mRNA levels to protein levels [3,4]. Unfortunately, most mRNAs and proteins could not be compared because the other ~70 interferon- and NF-κB-responsive genes reported by O’Donnell et al. [40] were not found by us.

We previously determined HEK293 responses to reovirus T1L infection [21]. That study identified and measured 2992 proteins at 24 hpi, 104 of which were up-regulated and 49 of which were down-regulated. Only 194 (~ 6.5%) of these same proteins were identified in the current study (Additional file 1: Figure S3). A small number of proteins were similarly regulated by both viruses in both cell types. Most proteins (159-187, depending upon how cutoffs were set) were non-regulated in both cell types, and no proteins were up-regulated in one cell but down-regulated in the other (Additional file 1: Figure S3C). Although there was only ~6.5% overlap between the 2 protein datasets, which could represent differences in the virus used and/or in the type of cell analyzed, many of the highly regulated pathways and processes were similar between the two experimental conditions. For example, regulation of interferon signaling, immunomodulation and responses to virus were highly up-regulated in both studies. Only 5 of the 194 host proteins identified and measured in both the T1L-infected HEK-293 and T3D-infected HeLa cells were significantly regulated. Of these, only 1 (STAT1), discussed in more detail earlier, was significantly regulated by both cell/virus conditions, being up-regulated 2.1-fold in T1L-infected HEK293 cells and up-regulated 3.2-fold in T3D-infected HeLa cells. One other protein (SCO1) was up-regulated 8.4-fold in T1L-infected HEK-293 cells but only moderately up-regulated (1.4-fold, Z-score = 1.088σ) in T3D-infected HeLa cells. SCO1 is a metallochaperone involved in copper homeostasis and found in the mitochondrial intermembrane space [70]. The SCO genes appear evolutionarily conserved [71] and have not yet been reported to have roles in virus replication. Two proteins were significantly up-regulated in one cell type but not regulated in the other. PRKCDBP, a potential tumor suppressor gene [72], and COMT, a degradative methyltransferase [73] that has been associated with cognitive deficits in herpes simplex type 1 virus infections [74], were up-regulated 2.3-, and 3.1-fold, respectively, in T1L-infected HEK-293 cells. However, the COMT ratio is based upon a single measured peptide so less reliable. One protein was significantly down-regulated in one cell type but not regulated in the other. PLCG1, involved in immune regulated signal transduction [75], was down-regulated 3-fold (I:M ratio of 0.33) in T3D-infected HeLa cells. Twenty six other proteins were moderately regulated (Z score 1.000 – 1.959σ, corresponding to ~ 0.9- – 2.2-fold up-regulation; or -1.000 – -1.959σ, corresponding to ~ 0.9- – 1.6-fold down-regulation [I:M ratios of 0.63 – 1.13) (Additional file 3: Table S2). Although measured values were not significant, one additional protein (C2orf43), a gene that has been associated with prostate cancer [76], was moderately down-regulated in both the T1L and T3D infections, with infected:mock values of .66 and 0.61, respectively. A few of these determined values are based upon 1 or 2 peptides. Therefore, additional work is needed to determine whether these apparent similarities and differences are real and virus- and/or cell-specific. We are currently analyzing T1L- and T3D-infected HeLa cells labeled by the iTRAQ reagent to simultaneously compare multiple virus types to mock and to use a complementary approach that may identify additional regulated proteins.

In summary, this non-biased global analysis has identified numerous host proteins significantly affected by reovirus T3D infection. These proteins complement others determined in other studies, fit within numerous inflammatory and innate immune pathways, and will provide the starting point for more detailed kinetic studies, such as initiated herein, as well as studies aimed at delineation of virus-specific pathways.

## Competing interests

The author declares no conflicts of interest.

## Supplementary Material

Additional file 1: Figure S1Gene ontology distribution of light-labeled proteins in indicated samples collected at 24 hpi from Mock- and T3D-infected HeLa cells; , nuclear-annotated proteins; , non-nuclear-annotated proteins. Total numbers of proteins and percentages indicated within each pie section. **Figure S2.** Additional top-ranked canonical pathways identified by IPA™. Red indicates highly up-regulated proteins, light green indicates down-regulated, grey represents not significantly affected, and white represents molecules known to be part of the pathway but not identified by SILAC. **Figure S3.** Comparison of reovirus-induced protein identifications and regulation in HEK293 cells [21] and in HeLa cells (this study). **A**, Venn diagram of numbers of identified proteins, and their overlap, from the 2 studies. **B**, Dot plot distributions of the Z-scores of each of the 194 proteins identified in both studies. **C**, Comparative distributions of regulated proteins, using Z-score category cutoffs of ± 1.960 (upper value in each cell) and ± 1.000 (lower *italicized* value in each cell). For example, 1 protein (STAT1) had a Z-score > 1.960 in both the HEK293 and HeLa cell lists and 2 proteins (STAT1 and cytochrome oxidase deficient homolog 1) had Z-scores > 1.000 in both the HEK293 and HeLa cell lists.Click here for file

Additional file 2: Table S1 All identified proteins and their associated # peptides, % coverage, Infected: Mock ratio, and calculated Z-scores a. Click here for file

Additional file 3: Table S2 Common proteins regulated in either T1L-infected HEK-293 or T3D-infected HeLa cells. Click here for file
