# Supplementary material for: HeLa cell response proteome alterations induced by mammalian reovirus T3D infection
Source: Virol J. 2013 Jun 21;10:202. doi: 10.1186/1743-422X-10-202 (PMC3847587; doi:10.1186/1743-422X-10-202)
Supplement: Additional file 1: Figure S1 — Gene ontology distribution of light-labeled proteins in indicated samples collected at 24 hpi from Mock- and T3D-infected HeLa cells; , nuclear-annotated proteins; , non-nuclear-annotated proteins. Total numbers of proteins and percentages indicated within each pie section. Figure S2. Additional top-ranked canonical pathways identified by IPA™. Red indicates highly up-regulated proteins, light green indicates down-regulated, grey represents not significantly affected, and white represents molecules known to be part of the pathway but not identified by SILAC. Figure S3. Comparison of reovirus-induced protein identifications and regulation in HEK293 cells [21] and in HeLa cells (this study). A, Venn diagram of numbers of identified proteins, and their overlap, from the 2 studies. B, Dot plot distributions of the Z-scores of each of the 194 proteins identified in both studies. C, Comparative distributions of regulated proteins, using Z-score category cutoffs of ± 1.960 (upper value in each cell) and ± 1.000 (lower italicized value in each cell). For example, 1 protein (STAT1) had a Z-score > 1.960 in both the HEK293 and HeLa cell lists and 2 proteins (STAT1 and cytochrome oxidase deficient homolog 1) had Z-scores > 1.000 in both the HEK293 and HeLa cell lists. [file 1743-422X-10-202-S1.pdf]

Supplementary Figure S1

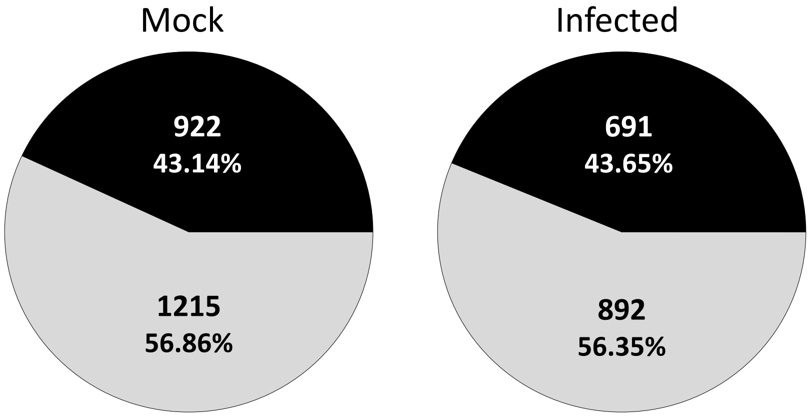

Figure S1. Gene ontology distribution of light-labeled proteins in indicated samples collected at 24hpi from Mock- and T3D-infected HeLa cells; ■, nuclear-annotated proteins; □, non-nuclear-annotated proteins. Total numbers of proteins and percentages indicated within each pie section.

Supplementary Figure S2. Top-ranked Ingenuity Canonical Pathways

Ingenuity Canonical Pathway	-log(p-value)
Interferon Signaling (see **Fig. 5**)	6.05E00
Glycogen Degradation II	3.97E00
PDGF Signaling	3.96E00
Glycogen Degradation III	3.71E00
Role of Pattern Recognition Receptors in Recognition of Bacteria and Viruses	3.56E00
Antigen Presentation Pathway	3.33E00
Prolactin Signaling	3.12E00
Protein Ubiquitination Pathway	3.03E00
EGF Signaling	2.97E00
Thrombopoietin Signaling	2.68E00
Glioma Invasiveness Signaling	2.62E00
T Cell Receptor Signaling	2.59E00
Activation of IRF by Cytosolic Pattern Recognition Receptors	2.54E00

Supplementary Figure S2-A: Glycogen degradation II

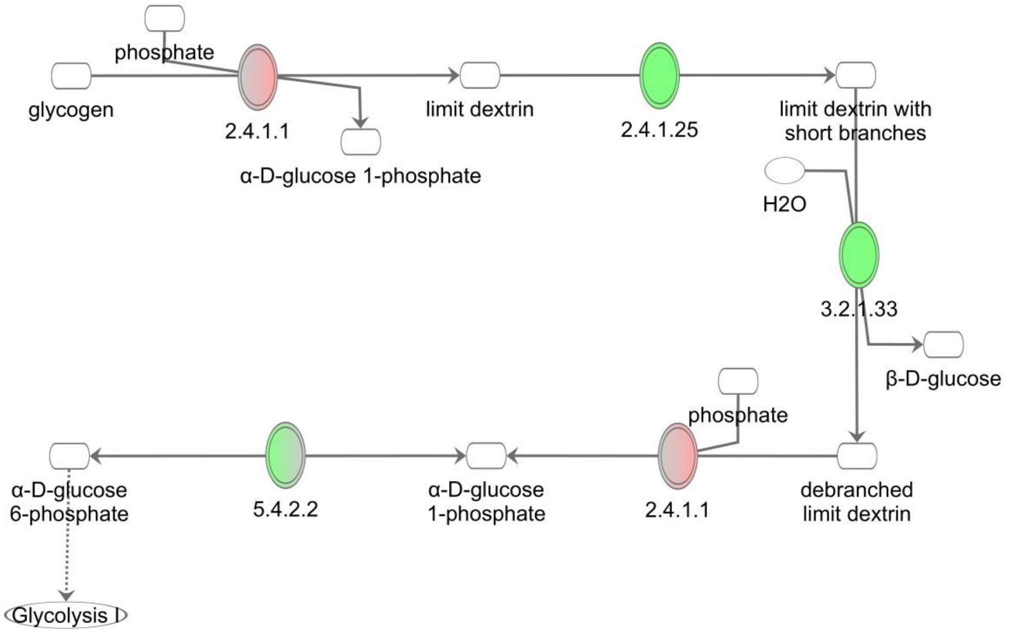

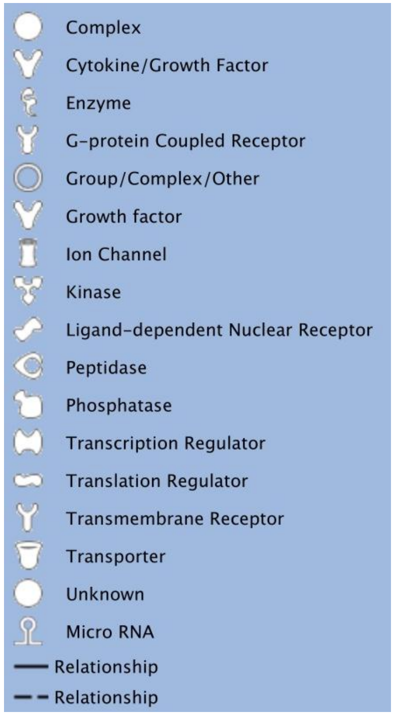

Supplementary Figure S2-B: PDGF signaling

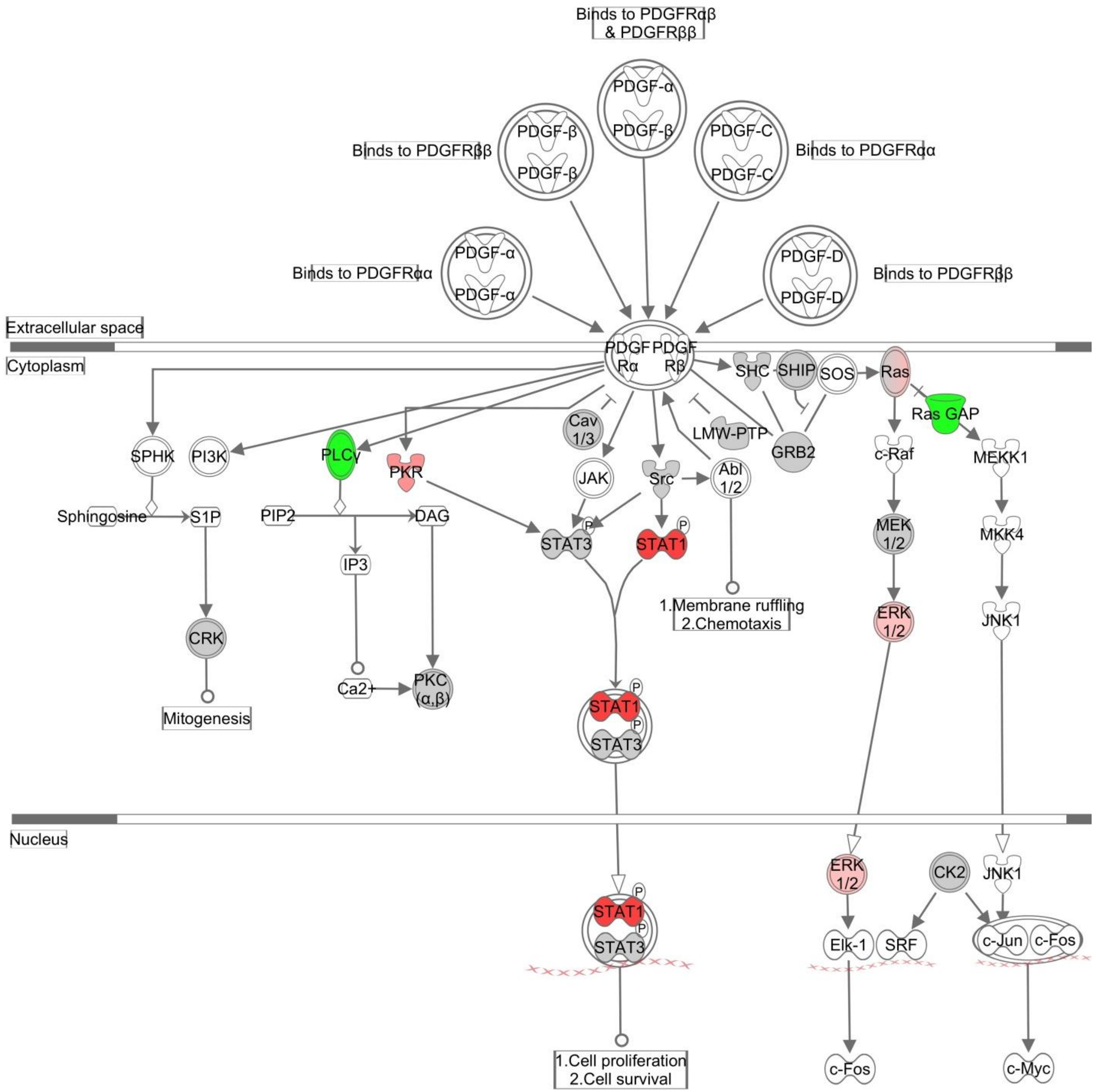

Supplementary Figure S2-C: Glycogen degradation III

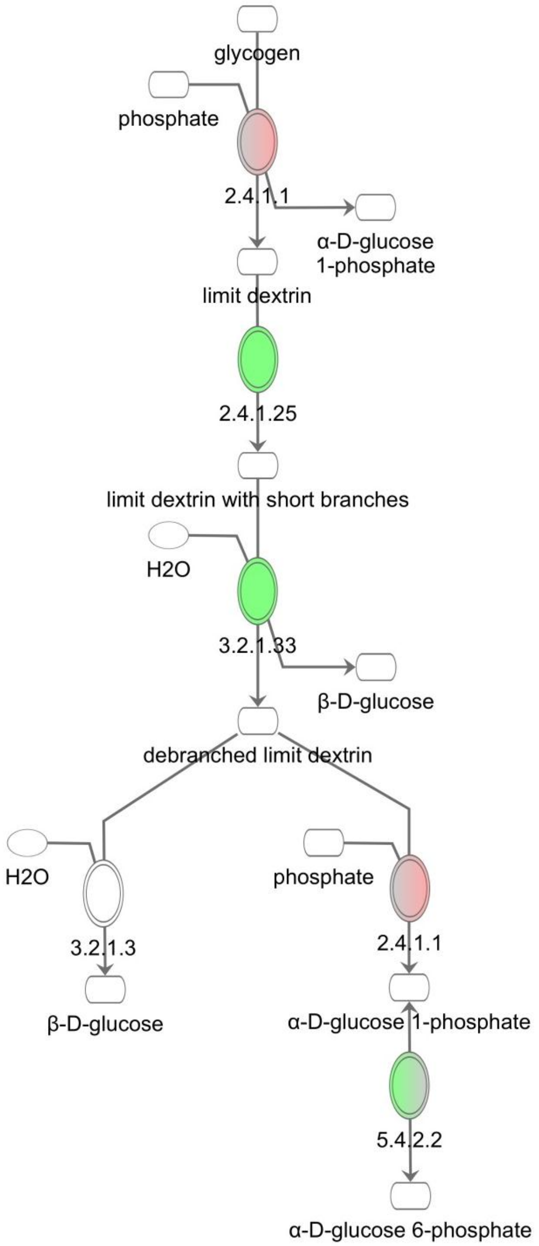

Supplementary Figure S2-D: Role of pattern recognition receptors in recognition of bacteria and viruses

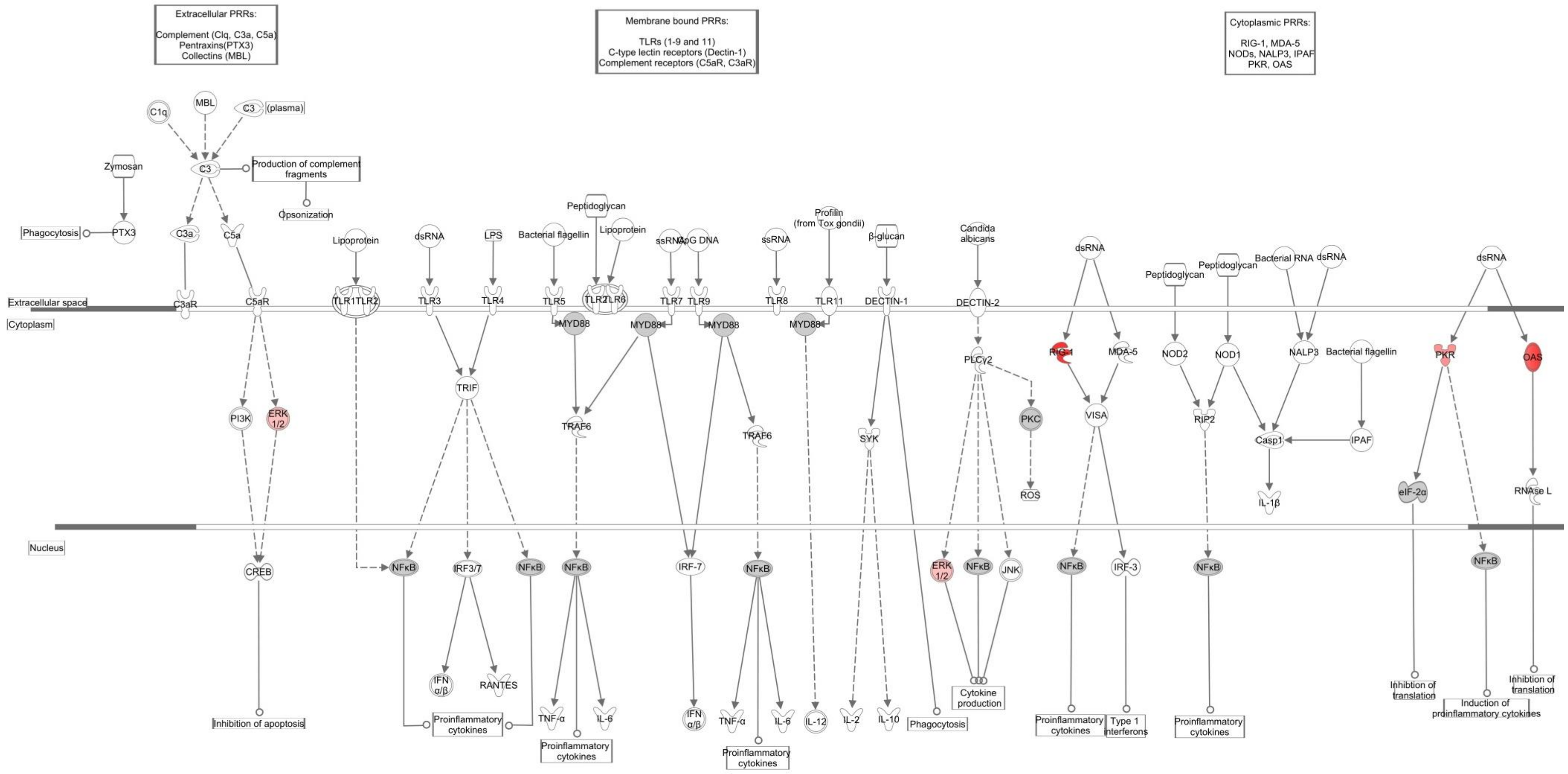

Supplementary Figure S2-E: Antigen presentation pathway

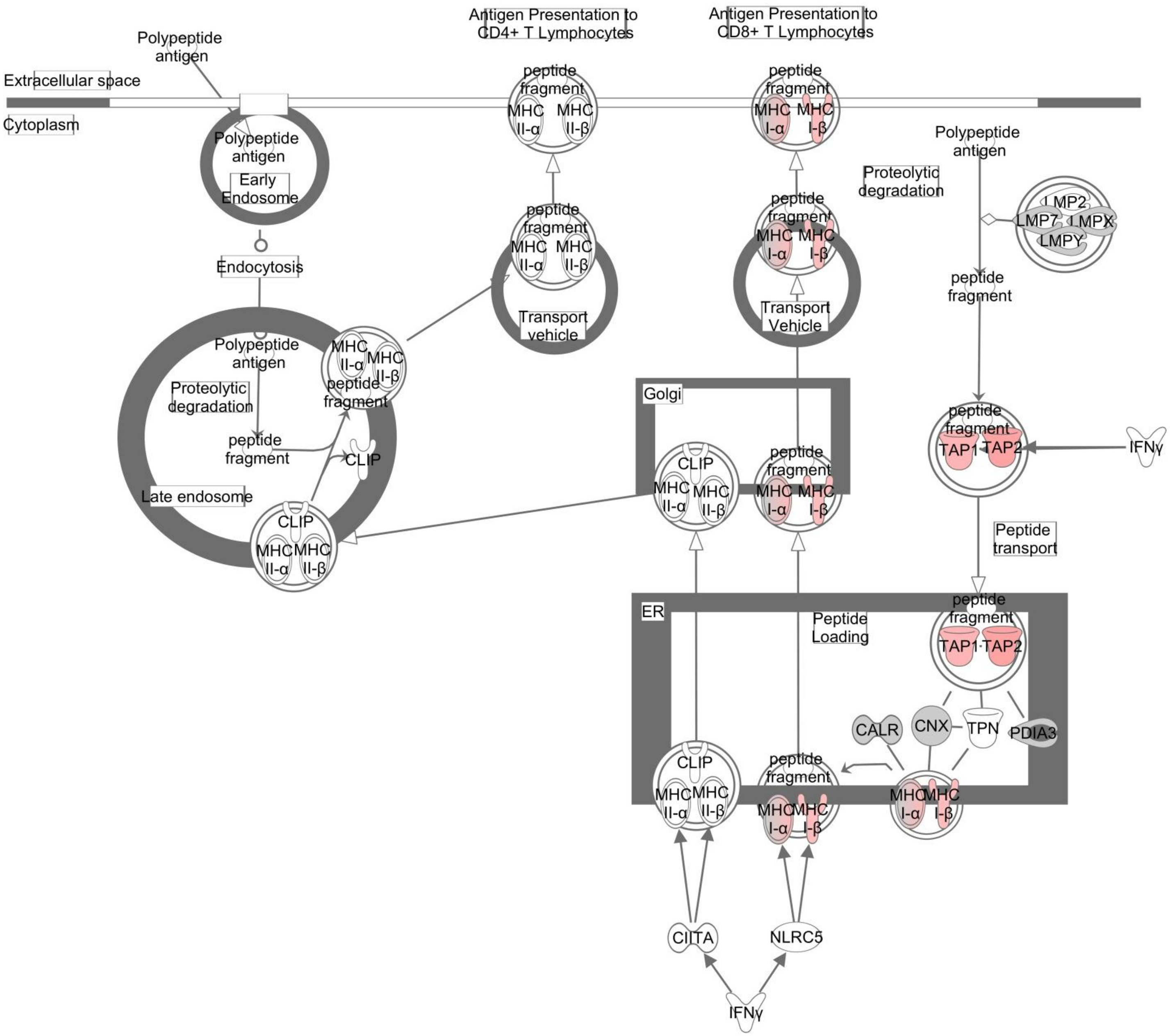

Supplementary Figure S2-F: Prolactin signaling

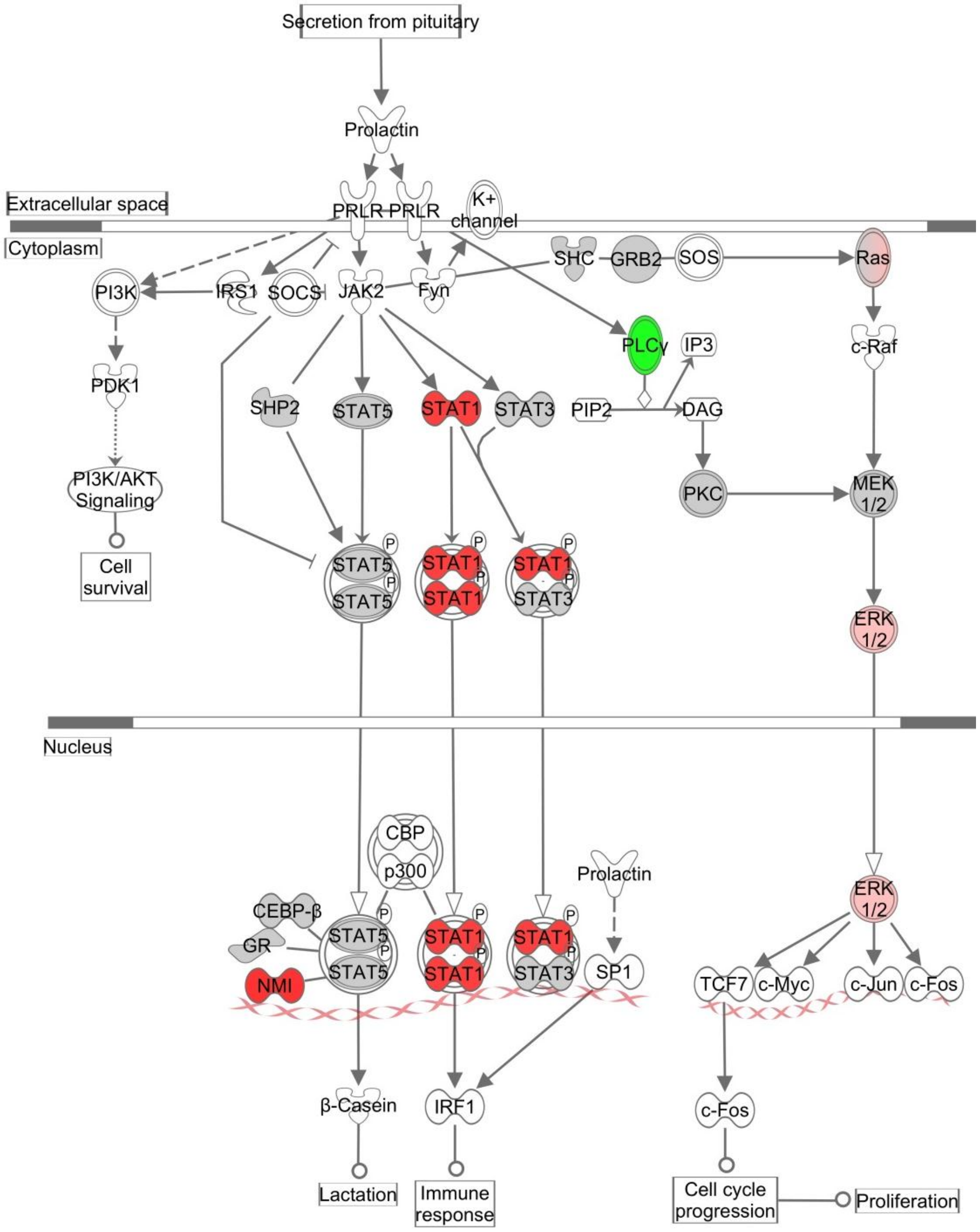

Supplementary Figure S2-G: Protein ubiquitination pathway

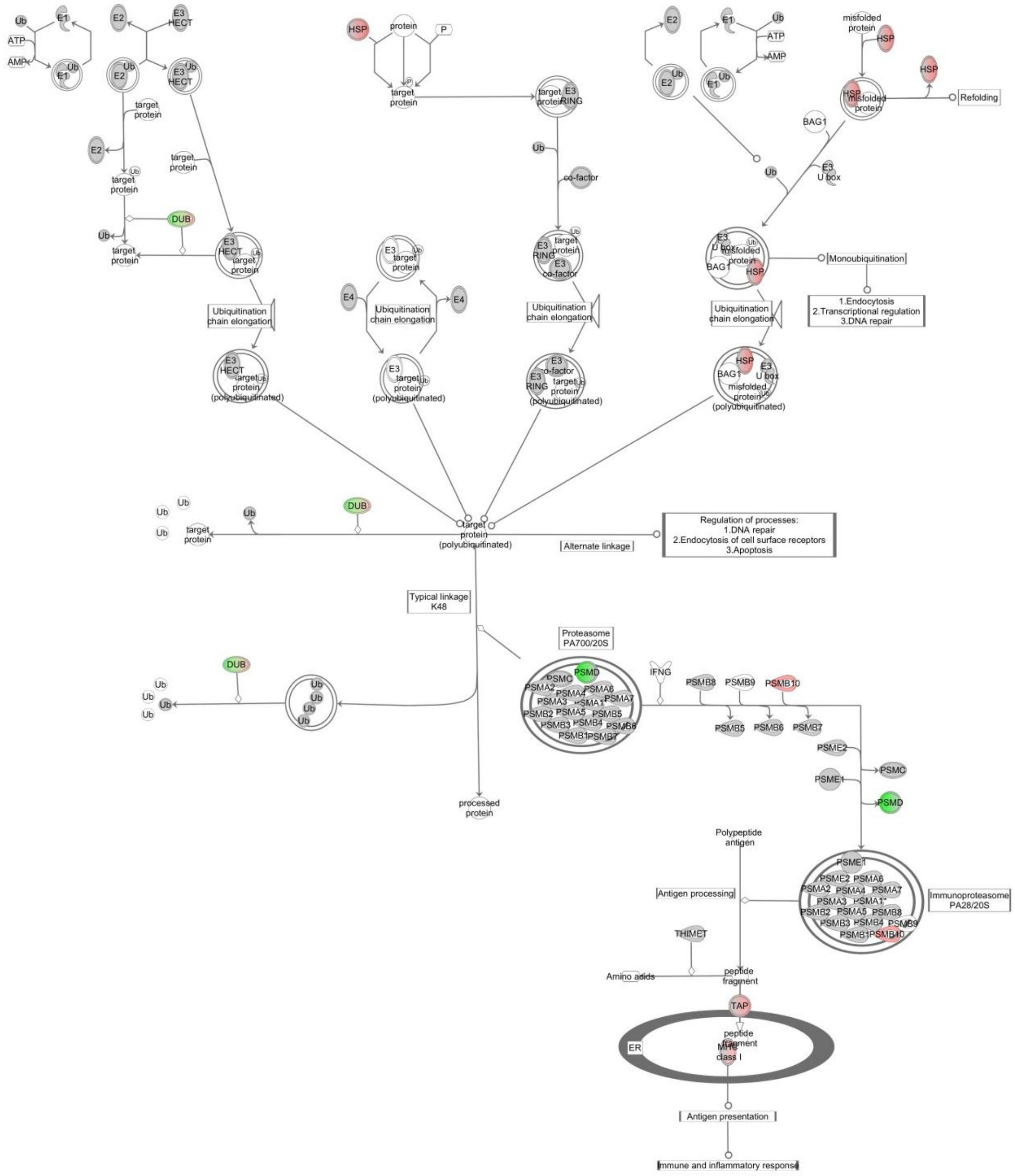

Supplementary Figure S2-H: EGF signaling

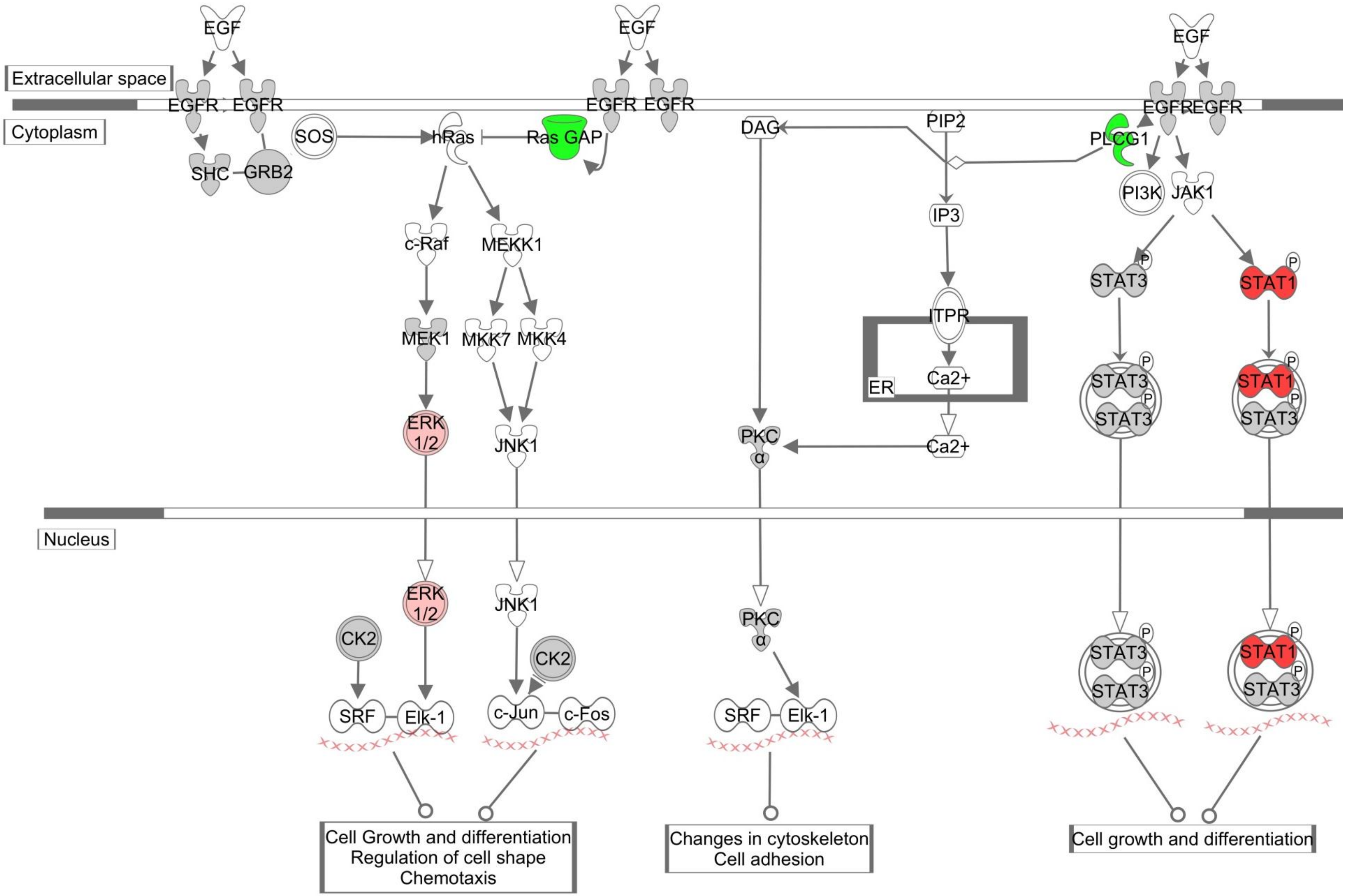

Supplementary Figure S2-I: Thrombopoietin signaling

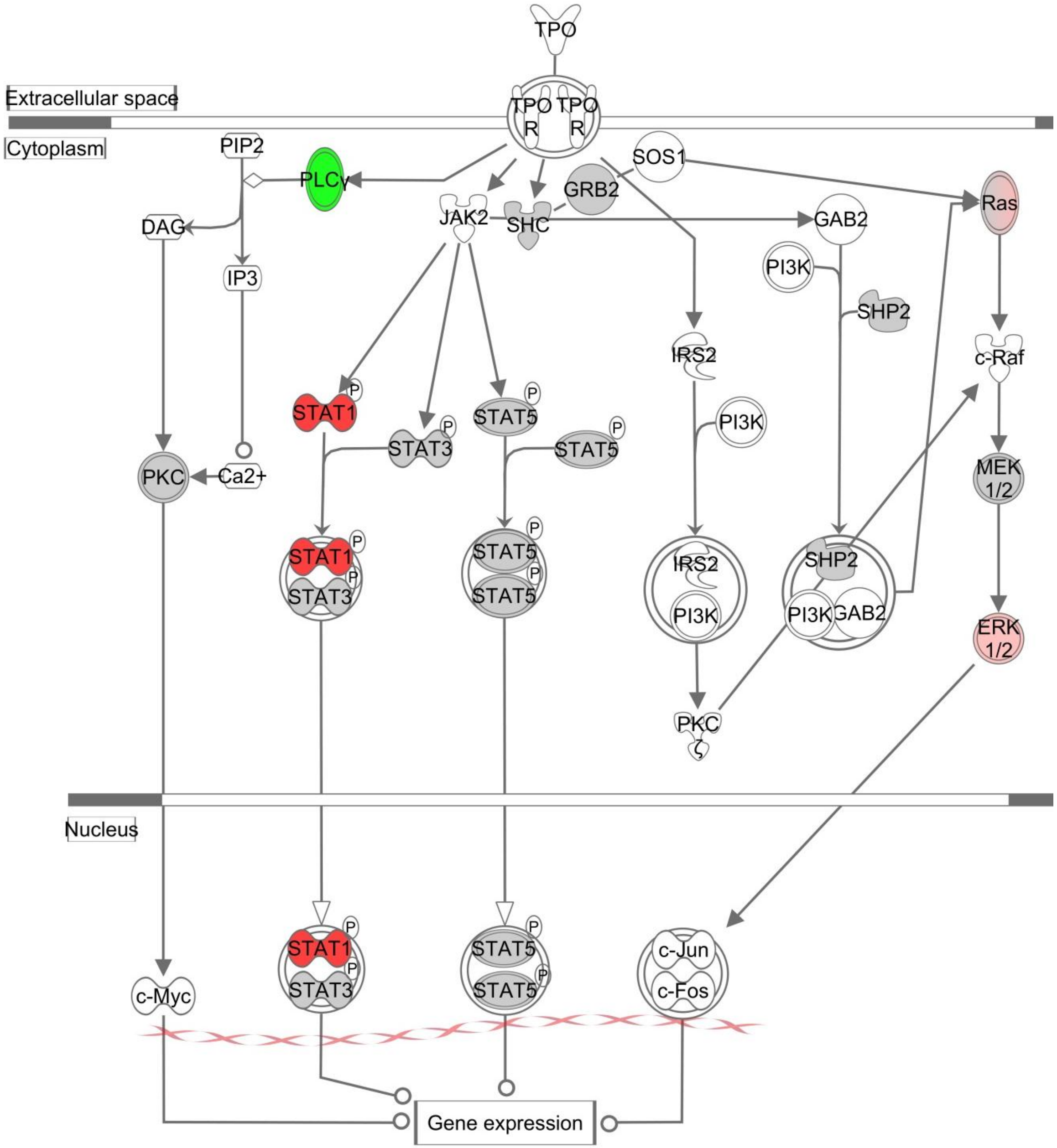

Supplementary Figure S2-J: Glioma invasiveness signaling

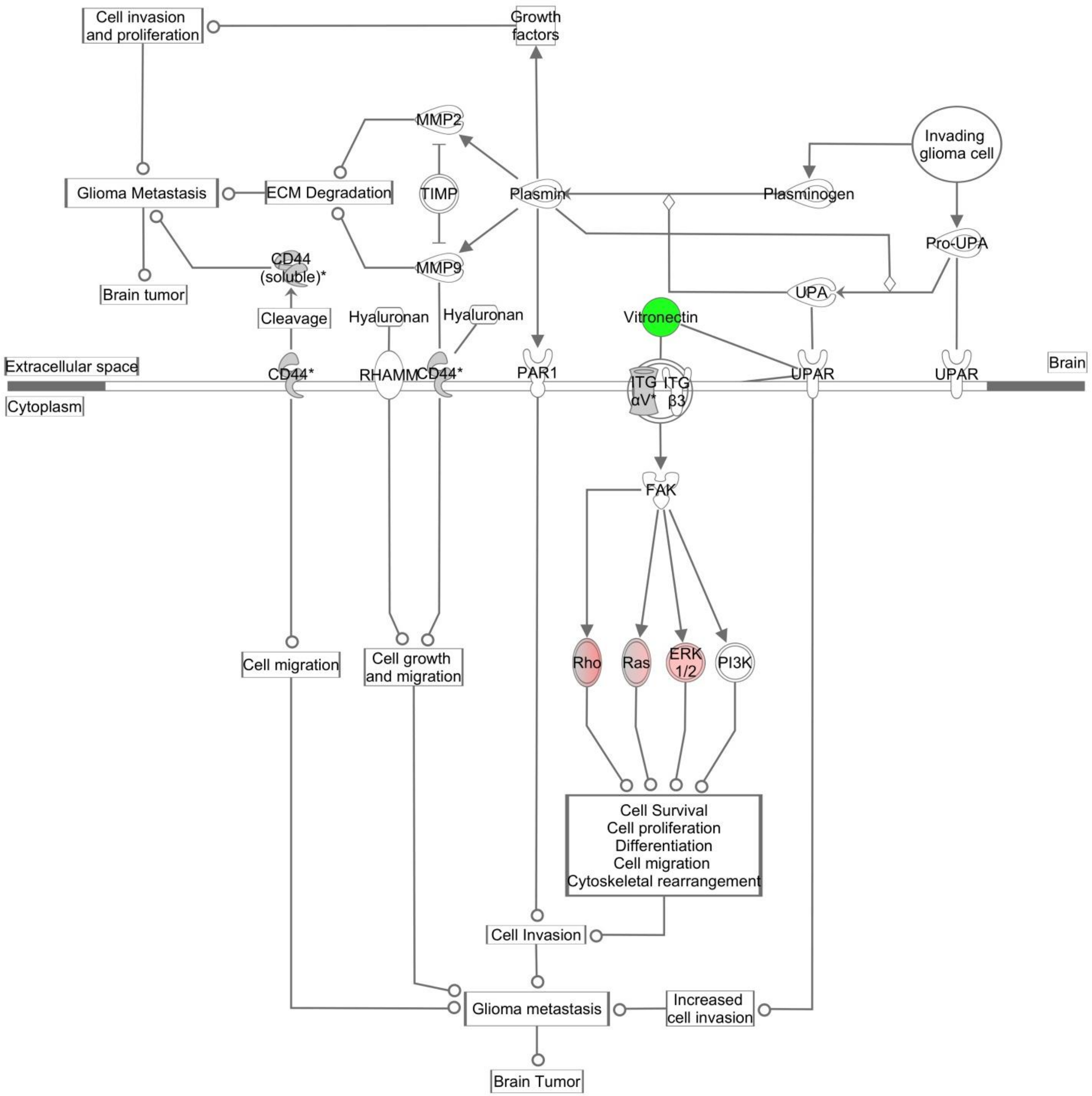

Supplementary Figure S2-K: T cell receptor signaling

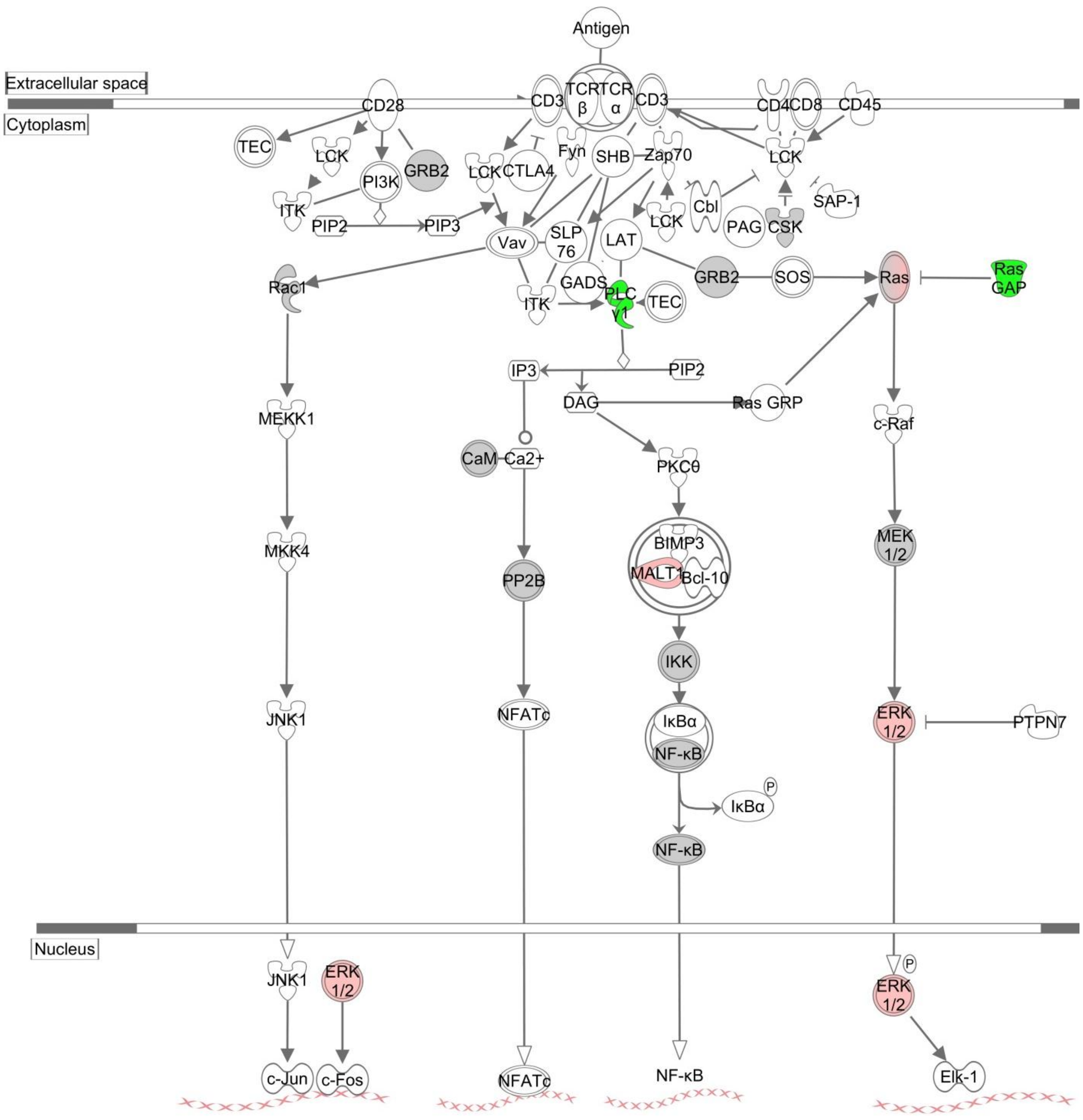

Supplementary Figure S2-L: Activation of IRF by cytosolic pattern recognition receptors

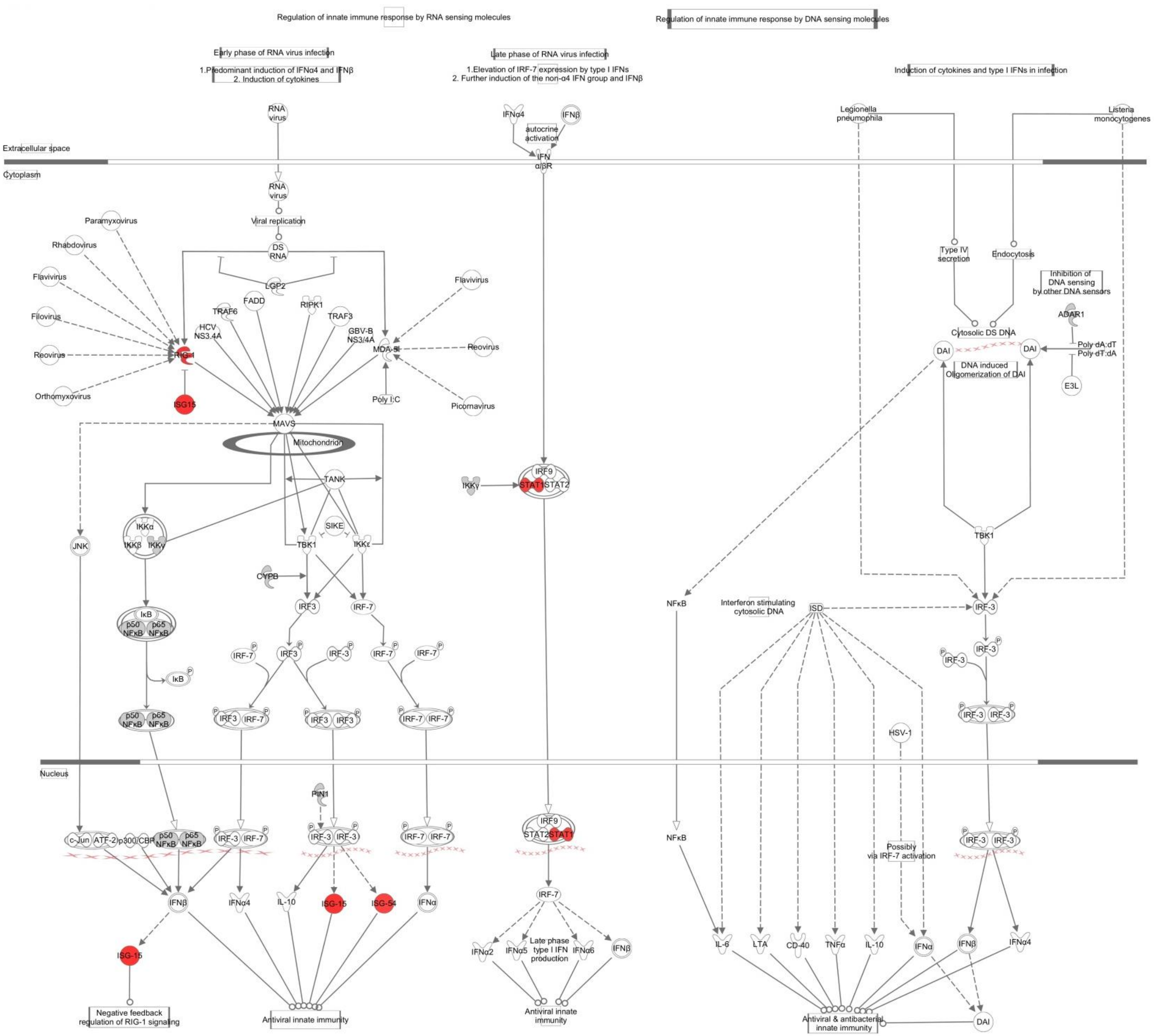

Supplementary Figure S3

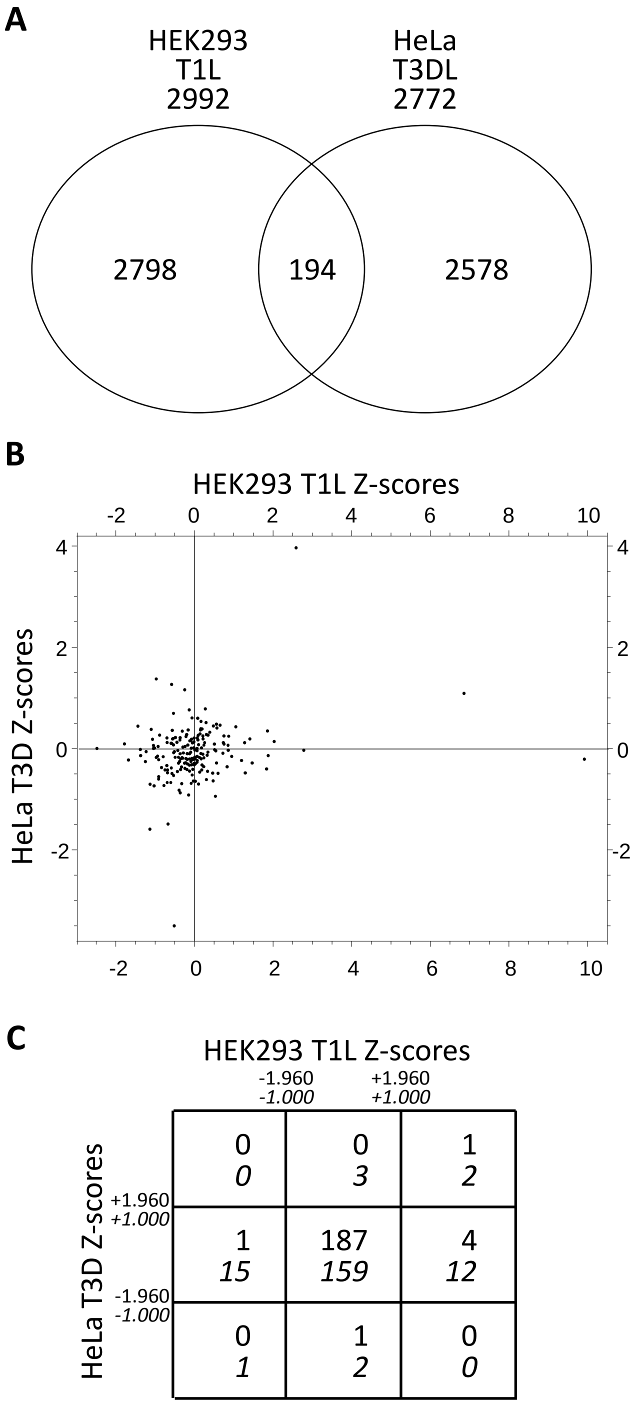

Figure S3. Comparison of reovirus-induced protein identifications and regulation in HEK293 cells [21] and in HeLa cells (this study). **A.** Venn diagram of numbers of identified proteins, and their overlap, from the 2 studies. **B.** Dot plot distributions of the Z-scores of each of the 194 proteins identified in both studies. **C.** Comparative distributions of regulated proteins, using Z-score category cutoffs of ± 1.960 (upper value in each cell) and ± 1.000 (lower *italicized* value in each cell). For example, 1 protein had a Z-score > 1.960 in both the HEK293 and HeLa cell lists and 2 proteins had a Z-score > 1.000 in both the HEK293 and HeLa cell lists.
